# Phosphorylation of the *Drosophila* Transient Receptor Potential Ion Channel Is Regulated by the Phototransduction Cascade and Involves Several Protein Kinases and Phosphatases

**DOI:** 10.1371/journal.pone.0073787

**Published:** 2013-09-09

**Authors:** Olaf Voolstra, Jonas-Peter Bartels, Claudia Oberegelsbacher, Jens Pfannstiel, Armin Huber

**Affiliations:** 1 Department of Biosensorics, Institute of Physiology, Universität Hohenheim, Stuttgart, Germany; 2 The Life Science Center, Universität Hohenheim, Stuttgart, Germany; University of Houston, United States of America

## Abstract

Protein phosphorylation plays a cardinal role in regulating cellular processes in eukaryotes. Phosphorylation of proteins is controlled by protein kinases and phosphatases. We previously reported the light-dependent phosphorylation of the *Drosophila* transient receptor potential (TRP) ion channel at multiple sites. TRP generates the receptor potential upon stimulation of the photoreceptor cell by light. An eye-enriched protein kinase C (eye-PKC) has been implicated in the phosphorylation of TRP by *in* *vitro* studies. Other kinases and phosphatases of TRP are elusive. Using phosphospecific antibodies and mass spectrometry, we here show that phosphorylation of most TRP sites depends on the phototransduction cascade and the activity of the TRP ion channel. A candidate screen to identify kinases and phosphatases provided *in vivo* evidence for an involvement of eye-PKC as well as other kinases and phosphatases in TRP phosphorylation.

## Introduction

Reversible protein phosphorylation regulates a multitude of biological functions in eukaryotes. Levels of protein phosphorylation are controlled by kinases that catalyze the phosphorylation of proteins at serine, threonine, and tyrosine residues, and phosphatases that remove phosphoryl groups from proteins. Protein phosphorylation mediates protein-protein interaction, regulates enzyme activity, controls subcellular localization, influences protein stability, and regulates the activity of ion channels. Eukaryotic protein kinases comprise a single protein superfamily sharing a common catalytic structure. They can be subdivided into eight different families (AGC, CaMK, Casein Kinase I, CMGC, STE, PTK, OPK, atypical and unknown kinases) according to their structural and functional properties [1]. In contrast to protein kinases that share a common catalytic structure, protein phosphatases have different structures, exhibit different catalytic mechanisms, and are subdivided into two major groups, the serine/threonine protein phosphatases (STPs) and protein tyrosine phosphatases (PTPs). STPs are further subdivided into the phosphoprotein phosphatase (PPP) and Mg^2+^- or Mn^2+^-dependent (PPM) families [2]. The PPP family of STPs comprises PP1, PP2A, and PP2B that are calcineurin- or Ca^2+^-regulated and have been implicated in the regulation of several biological processes and signal transduction pathways. PPP family STPs share a limited number of conserved catalytic subunits. However, the diverse physiological roles are accomplished by a large number of regulatory domains. The PPM family comprises PP2c and mitochondrial pyruvate dehydrogenase phosphatase that are Mg^2+^-dependent. Data on the biological function of PPM family STPs is scarce. PTPs share the common catalytic motif, Cys-X5-Arg [3], and can be subdivided into classical PTPs comprising receptor and cytoplasmic PTPs, dual specificity phosphatases, and low molecular weight phosphatases. Taken together, about 251 protein kinases and 86 protein phosphatases have been identified in the *Drosophila* genome [4].

The visual transduction cascade in *Drosophila* photoreceptor cells is a G protein-mediated, phospholipase Cβ-dependent signaling pathway that is activated through absorption of a photon by rhodopsin and terminates in the opening of the cation channels TRP and TRPL. Known kinases and phosphatases of proteins of the *Drosophila* phototransduction cascade comprise eye protein kinase C (eye-PKC) [5]–[7], PP2A [8], rhodopsin kinase (GPRK1) [9], and retinal degeneration C (RDGC) [10]. Members of the phototransduction cascade are tethered to the inactivation no afterpotential D (INAD) scaffolding protein to form a supramolecular complex. INAD tethers the transient receptor potential (TRP) ion channel, phospholipase Cβ (PLC), neither inactivation nor afterpotential C (NINAC), and eye-PKC [11]–[19]. Since eye-PKC is a member of the INAD signaling complex, it has been proposed that eye-PKC phosphorylates other members of the complex. Indeed, eye-PKC phosphorylates TRP and INAD *in vitro* [6], [7]. *Drosophila* PP2A encoded by the *mts* locus has been indirectly shown to dephosphorylate INAD [8]. Incubation of isolated INAD signaling complexes with [γ-^32^P]ATP and head extracts from heterozygous *mts* mutant flies resulted in considerably elevated INAD phosphorylation levels as compared to incubation with head extracts from wild type control flies [8]. However, the *mts* mutation had no obvious effect on TRP phosphorylation. A heterozygous *mts* mutation rescued the prolonged deactivation phenotype of the visual response in *inaC^P209^* flies lacking eye-PKC [8]. Like most G protein-coupled receptors, *Drosophila* rhodopsin becomes phosphorylated upon light activation and this phosphorylation is removed when the receptor is reconverted to its inactivated state [20]–[22]. Rhodopsin phosphorylation is mediated by the rhodopsin kinase encoded by the *gprk1* gene [23]. GPRK1 is expressed in photoreceptor cells and functions in modulating the amplitude of the visual response. The retinal degeneration C (*rdgC*) locus encodes the corresponding phosphatase required for rhodopsin dephosphorylation [10]. The *rdgC* mutant fly exhibits severe light-dependent retinal degeneration and defective deactivation kinetics of the visual response. Expression of a truncated rhodopsin protein lacking the putative phosphorylation site targeted by RDGC in an *rdgc* mutant background rescued both the retinal degeneration and the slow deactivation phenotype [10].

Recently, we reported the identification of 21 TRP phosphorylation sites [24]. These sites are embedded within consensus sequences for several protein kinases but it is elusive which kinases and phosphatases contribute to TRP phosphorylation *in vivo*. Here, we present a detailed analysis of the dependence of the TRP phosphorylation sites on the phototransduction cascade and on light. Using two phosphospecific antibodies, we determined the occupancy of two phosphorylation sites to be at most 56% (Thr849) and 34% (Thr864) in the light, and we present the results of a candidate screen to identify kinases and phosphatases involved in regulating the phosphorylation state of the *Drosophila* TRP protein. We provide *in vivo* evidence that eye-PKC is one but not the only protein kinase involved in TRP phosphorylation.

## Materials and Methods

### Fly Stocks

The following strains and mutants of *Drosophila melanogaster* were used: *w* Oregon R, *yw*;;*trp^P343^* [25], *w*;;*trp^P365^* [26], *yw;;ninaE^17^* [27], *w*;*Gαq^1^* [28], *w*,*norpA^P24^* [29], *w*;*inaC^P209^* [30], *w*;+;*trp^D621G^*,*trp^P343^* [31], *w*;*rdgC^306^* [20]. A list of flies used for the candidate screen to identify phosphatases and kinases of TRP is shown in Table S2 of the supplementary section. All flies were obtained from Bloomington Stock Center, Indiana, unless noted otherwise. Flies were reared at 25°C. For experiments, 1 to 5 day-old-flies were used unless noted otherwise. Flies were illuminated with a 30-watt white light fluorescent lamp, 2000 lux. For analysis of TRP phosphorylation by mass spectrometry, flies were illuminated or kept in the dark over night before they were subjected to immunoprecipitation. To investigate the phosphorylation state of TRP at Thr^849^ and Thr^864^ in different mutants of the phototransduction cascade using phosphospecific antibodies, flies were dark-adapted for 12–18 h and were then illuminated for 1 h or *vice versa* before they were subjected to Western blot analysis. To screen candidate mutants for an alteration in TRP phosphorylation at Thr^849^ and Thr^864^, flies were light- or dark-adapted for 12–18 h and were then subjected to the opposite light condition for 1 h prior to Western blot analysis. Dark-kept flies were dissected under dim red light (Schott RG665, LED light source Nikon C-LEDS), and flies kept in white light were dissected under this light condition.

### Immunoprecipitation of TRP from Fly Heads for Mass Spectrometry and Western Blotting

Flies used for immunoprecipitation were wild type, *norpA^P24^*, and *trp^P365^*/+. Immunoprecipitation was carried out as described before using a polyclonal α-TRP antibody [24]. For estimation of phosphate occupancy at Thr849 and Thr864, phosphorylated TRP was precipitated using α-pT849 and α-pT864 antibodies.

### Mass Spectrometry (MS) Analysis

In-gel digestion was performed as previously described [24]. HPLC and electrospray mass spectrometry were carried out as described [24] with modifications. Gradient elution was performed from 1% acetonitrile to 50% acetonitrile in 0.1% formic acid within 120 min. Survey spectra were detected in the range of m/z = 250–1800. Mass spectrometry data analysis was carried out as described [24]. Quantification of light-dependent TRP phosphorylation sites was accomplished by a label-free nano-HPLC-MS approach. For each light condition (light or dark) and each genotype (wild type, *norpA^P24^*, *trp^P365^*/+), six independent experiments were carried out. Immunoprecipitated TRP from three of these experiments was digested with trypsin and TRP from the other three experiments was digested with chymotrypsin. For each experiment, two technical replicates were carried out. Thermo raw files of the acquired nano-LC-MS/MS runs were processed and analyzed using Progenesis LC-MS software (version 3.1.4, Nonlinear Dynamics, UK). The LC-MS runs in each dataset were automatically aligned using one LC-MS run as reference. Two-dimensional maps (m/z versus retention time) of each run were generated, aligned to the reference run, and used to find a common set of feature outlines (signals defined by m/z ratio and retention time). Peptide abundance values were subsequently calculated as the sum of the peak areas within the isotope boundaries of the feature. Abundance values for each feature were normalized to account for different amounts of sample or run-to-run variations. The normalization process implemented in the Progenesis LC-MS software is based on the assumption that most features show no change in abundance in a given experiment. For each feature, the software calculates a quantitative abundance ratio between the run being normalized and the reference run. The normalization factor for each run is then calculated from the distribution of the log of the abundance ratios using a recursive median approach. Further details about the calculation of peptide abundances and the normalization process in the Progenesis LC-MS software can be found at the company’s website (http://www.nonlinear.com/support/progenesis/lc-ms/faq/). Before quantification, the number of features was reduced to signals with a charge state of +2, +3, +4, and +5. TRP phosphopeptides were identified by means of a Mascot generic file created by the Progenesis LC-MS software using the same search parameters as described previously [24]. For relative quantification of phosphopeptide abundances, the mean phosphopeptide abundances from nine LC-MS runs of each condition were calculated and the higher value was set to 100%. Only phosphopeptides that were present in all biological and technical replicates of at least one light condition were considered for quantification. An unpaired student’s t-test was performed for statistical evaluation of the quantification results.

### Generation of Phosphospecific Antibodies

In order to produce antisera, two phosphopeptides, NH_2_-CGRKK(pT)QKGD-CONH_2_, comprising pT849, and NH_2_-CARKN(pT)FASD-CONH_2_, comprising pT864, were synthesized and injected into rabbits (Pineda antibody service). Affinity purification was carried out as described previously [24].

### Immunocytochemistry

Immunocytochemistry was carried out as described previously [32], with modifications. α-TRP (MAb83F6, Developmental Studies Hybridoma Bank), α-pT849, and α-pT864 were used as primary antibodies. Secondary antibodies were α-mouse Cy5 (Dianova), α-rabbit AlexaFluor 680 (LifeTechnologies), α-mouse AlexaFluor 488 (LifeTechnologies) and AlexaFluor 546-coupled phalloidin (LifeTechnologies).

### Quantitative Western Blot Analysis

1D gel electrophoresis and protein transfer onto nitrocellulose membranes (Roth) was carried out as described previously, with modifications [24]. Primary antibodies used were the same as for immunocytochemistry. α-rabbit IgG and α-mouse IgG coupled to horse radish peroxidase (Sigma) were used as secondary antibodies. The chemiluminescence reagent was prepared as follows: Solution I, 0.04% (w/v) luminol (Sigma) in 0.1 M Tris (pH 8.6). Solution II, 0.11% (w/v) para-hydroxy coumaric acid (Sigma). Solution III, 30% hydrogen peroxide. 2 ml solution I, 200 µl solution II, and 0.6 µl solution III were mixed and membranes were preincubated for 2 min prior to signal detection in an XRS+ documentation system (Biorad). TRP and phospho-TRP amounts were quantified with the Image Lab 4.0 application (Biorad) by determining the integrated density of each protein band, i.e. the sum of the pixel intensities in the protein band. For normalization, the values obtained using phosphospecific antibodies were divided by the TRP value of the same sample. Specificity of α-pT849 and α-pT864 antibodies was tested as shown in figure S2A. Linearity of signal intensity and amount of TRP present in the sample was confirmed as described in figure S2B.

### Candidate Screen to Identify TRP Kinases and Phosphatases

Candidate mutants in kinases and phosphatases with an eye mRNA signal of greater or equal to 70 according to the flyatlas gene expression database (http://flyatlas.org) were purchased from Bloomington Stock Center. Flies were light- or dark-adapted for 12–18 h and were then switched to the opposite light condition for 1 h before heads were subjected to Western blot analysis using the two phosphospecific antibodies.

## Results

### Quantification of Light-dependent TRP Phosphorylation Sites

**Figure 1:**
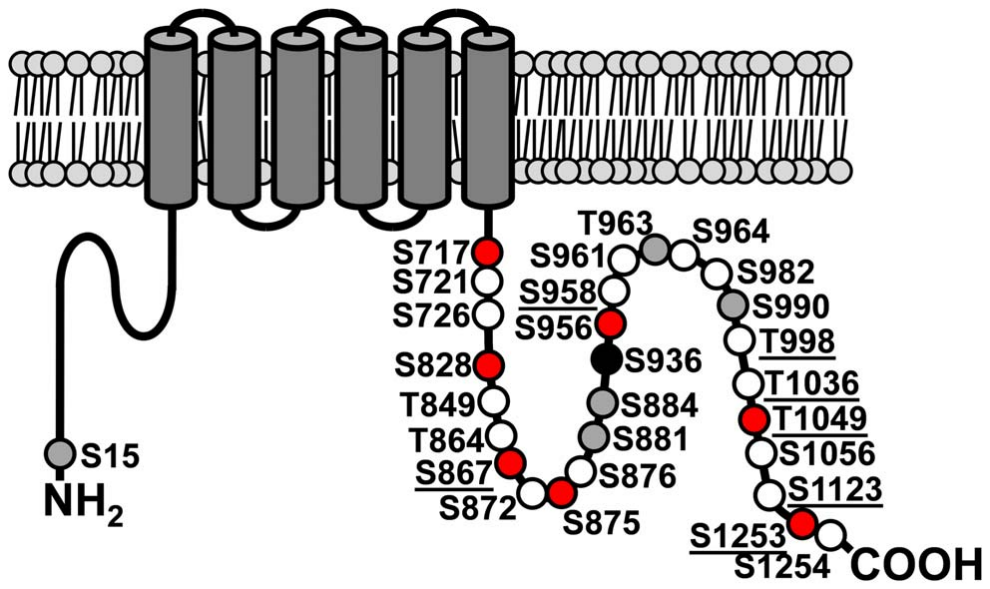
Cartoon of the TRP protein showing its phosphorylation sites. Circles represent TRP phosphorylation sites as revealed in this study and in [24]. White circles, sites that were predominantly phosphorylated in the light. Black circle, site that was predominantly phosphorylated in the dark. Grey circles, sites that were not light-dependently phosphorylated. Red circles, phosphorylation sites that were located on peptides that did not meet the criteria for quantification. Sites that were newly identified in this study are underscored.

**Figure 2:**
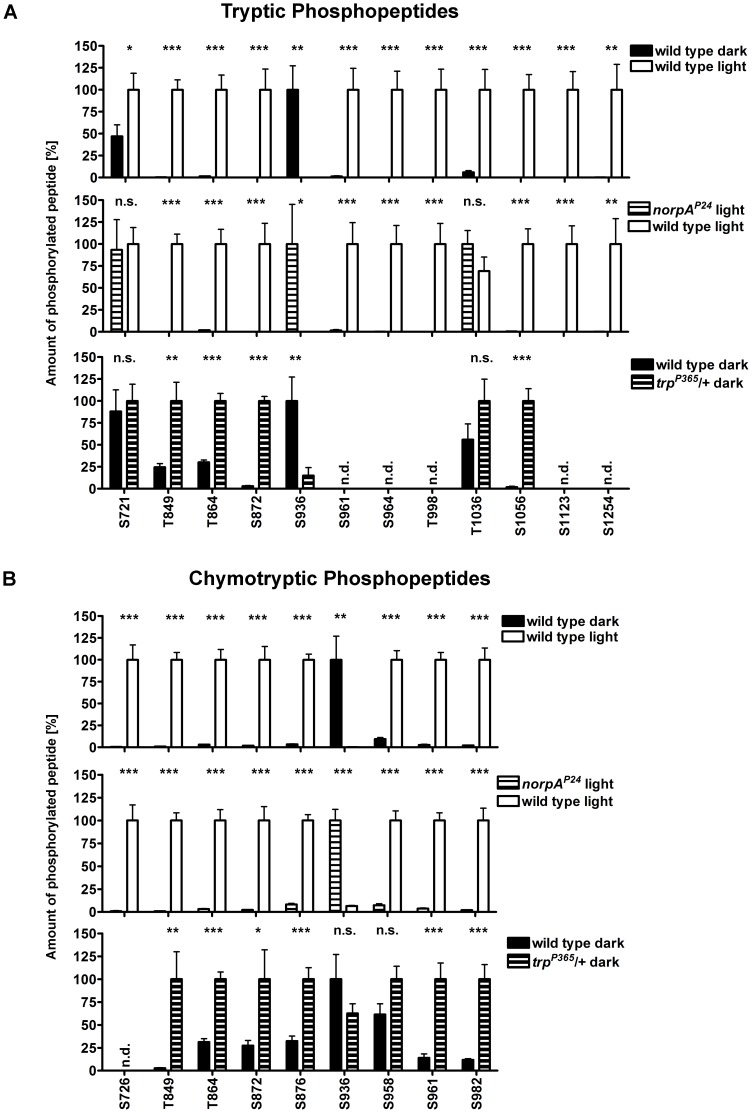
MS-based comparison of TRP phosphorylation. Wild type, *norpA^P24^*, and *trp^P365^*/+ flies were illuminated or kept in the dark prior to immunoprecipitation using an α-TRP antibody. Three independent experiments and two technical replicates for each genotype and light condition were analyzed by mass spectrometry. (A) Results obtained from tryptic peptides and (B) from chymotryptic peptides. Error bars indicate SEM. *, P<0.05; **, P<0.01; ***, P<0.001; n.s., not significant; n.d., not detected (i.e. did not meet the criteria for quantification).

Previously, we used a phosphospecific antibody directed against TRP phosphorylated at Ser936 and showed that dephosphorylation of Ser936 depended on the phototransduction cascade. Ser936 was the only site that was dephosphorylated in the light and became phosphorylated in the dark [24]. To obtain a broader view of the effect of mutations in central components of the phototransduction cascade on TRP phosphorylation, we conducted a quantitative mass spectrometry approach using two fly strains, *norpA^P24^* and *trp^P365^*. The *norpA^P24^* mutant is blind because it lacks functional phospholipase C, the central effector enzyme of the *Drosophila* phototransduction cascade that hydrolyzes PIP_2_ to yield IP_3_, DAG, and protons. The *trp^P365^* mutant expresses a constitutively active TRP channel and shows age-dependent degeneration of photoreceptor cells due to sustained Ca^2+^ influx. To ensure that photoreceptors were not degenerated in the flies used for mass spectrometry analysis, 1 to 2 day-old heterozygous *trp^P365^* flies (*trp^P365^*/+) were used. Flies were light- or dark-adapted over night and TRP extracted from fly heads was purified by immunoprecipitation and SDS PAGE. The coomassie-stained TRP band was in-gel digested with either trypsin or chymotrypsin and subjected to liquid chromatography tandem mass spectrometry (LC-MS/MS). Identification and quantification of phosphopeptides from wild type flies yielded similar results as before [24] except that we identified seven new phosphorylation sites in addition to the previously described 21 TRP phosphorylation sites [24] (Fig. 1). Identification of additional sites may result from using altered HPLC conditions (peptide elution over 120 min instead of 60 min) that allowed better separation of peptides prior to mass spectrometry. The newly discovered phosphorylation sites, Ser867, Ser958, Thr998, Thr1036, Thr1049, Ser1123, and Ser1253 were unequivocally identified by fragmentation spectra (Fig. S1). These sites are located in the C-terminal portion of TRP and together with the previously identified 20 C-terminal phosphorylation sites show that this region of the ion channel is highly phosphorylated at multiple sites whereas only a single site is present in the N-terminal region and no phosphorylation sites were identified in the transmembrane region (Fig. 1). TRP phosphopeptides were quantified by a label-free approach using the Nonlinear Dynamics Progenesis LC-MS software suite for chromatographic alignment, deisotoping, normalization, and peak area determination of MS1 features (peptides) over multiple LC-MS/MS runs. Only singly phosphorylated peptides on which the phosphorylation site could be unambiguously assigned and which were present in all LC-MS runs of at least one light condition were considered for quantification. When more than one peptide variant was available for a particular phosphorylation site, the one with the highest averaged normalized abundance was chosen. Phosphopeptides that were used for quantification are listed in table S1. Ser721, Ser726, Thr849, Thr864, Ser872, Ser936, Ser958, Ser961, Ser964, Thr998, Thr1036, Ser1123, and Ser1254 exhibited elevated phosphorylation in the light, whereas a single site, Ser936, displayed elevated phosphorylation in the dark. TRP phosphorylation in light-adapted *norpA^P24^* flies differed markedly from light-adapted wild type flies (Fig. 2A, B, middle panels). It rather resembled the situation in dark-adapted wild type flies. Except for the tryptic peptides containing Ser721 and Thr1036, all sites that were up-regulated in wild type flies in the light displayed significantly less phosphorylation in the light-adapted *norpA^P24^* flies, while Ser936, which becomes dephosphorylated in the light in wild type flies showed a high level of phosphorylation in the *norpA^P24^* mutant. These results show that for most sites phospholipase Cβ is necessary to trigger TRP phosphorylation (or dephosphorylation in the case of Ser936) in the light. In the *trp^P365^*/+ flies, the retrieval rate of phosphopeptides from tryptic and chymotryptic digests was lower than in the other flies analyzed. Overall, TRP phosphorylation in dark-adapted *trp^P365^*/+ flies differed markedly from TRP phosphorylation in dark-adapted wild type flies. TRP phosphorylation in dark-adapted *trp^P365^*/+ flies rather resembled the situation found in light-adapted wild type flies. Taken together, these results suggest that phosphorylation and dephosphorylation of most light-dependent TRP phosphorylation sites depend on phospholipase Cβ and on TRP ion channel activity.

### Phosphorylation of Thr849 and Thr864 is Triggered by the Phototransduction Cascade

According to predicted kinase consensus sequences and due to our findings showing that some sites become phosphorylated in the light while Ser936 even shows enhanced phosphorylation in the dark, it is likely that different kinases and phosphatases are involved in TRP phosphorylation. For further analysis, we focused on two sites, Thr849 and Thr864, that displayed a highly significant difference between light- and dark-adapted flies with much stronger phosphorylation in the light. Thr849 was predicted to be a protein kinase C phosphorylation site and Thr864 to be a protein kinase G phosphorylation site (NetPhosK phosphorylation site prediction tool). For a detailed analysis of TRP phosphorylation at Thr849 and Thr864, and for performing a screen to identify kinase(s) and phosphatase(s) of these sites, we generated two phosphospecific antibodies, α-pT849 and α-pT864, that specifically detected TRP phosphorylated at Thr849 or at Thr864, respectively. The specificity of the antibodies for the phosphorylated form of these sites was confirmed by dot blot analyses of the phosphopeptides used to generate the antibodies and the corresponding non-phosphorylated peptides (Fig. S2A). We also show that the antibodies detected only the phosphorylated Thr residue in its specific amino acid sequence context but not any phosphorylated Thr as the antibodies showed no cross-reactivity with other phosphopeptides (Fig. S2A).

**Figure 3:**
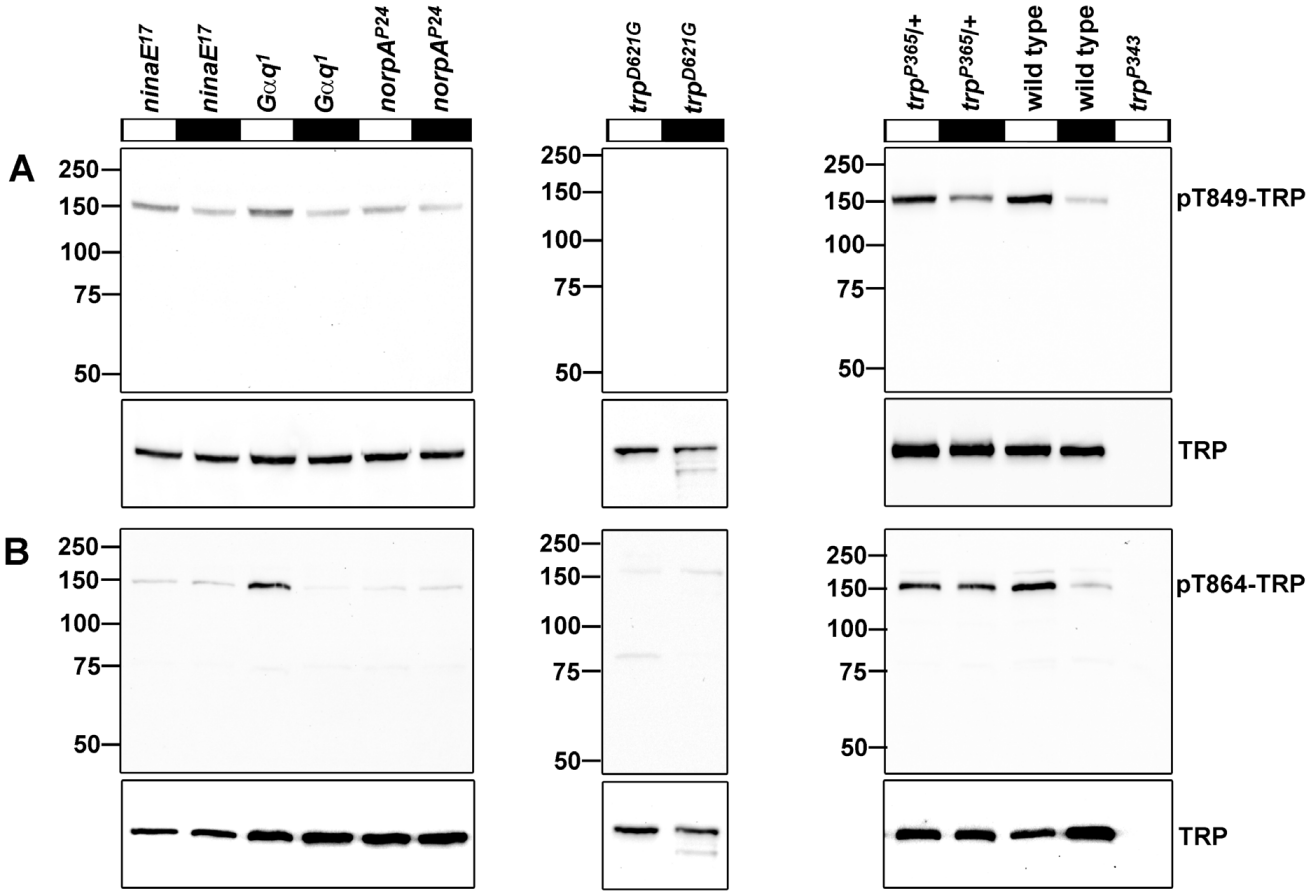
TRP phosphorylation at Thr849 and Thr864 in different mutants of the phototransduction cascade. Flies were illuminated for 12–18 h and then kept in the dark for 1 h (black bars) or *vice versa* (white bars) before they were subjected to Western blot analysis using α-pT849 (A) and α-pT864 (B) antibodies. The lower panels show Western blots probed with α-TRP antibody to reveal the amount of TRP present in the samples. Molecular mass markers (in kilodalton) are indicated to the left.

In order to demonstrate that the phosphospecific antibodies can be used to assess the phosphorylation of Thr849 and Thr864 quantitatively, we tested the linearity of the obtained signal intensities with the two phosphospecific antibodies by Western blot analysis using protein extracts containing different amounts of the TRP protein. To ensure equal concentrations of all other proteins, we mixed extracts from wild type fly heads with extracts from *trp^P343^* fly heads such that the amount of TRP was different while the total protein amount loaded on the gel was constant (3 heads). In the analyzed range (TRP from 3 to 0.375 heads), we observed good correlation (TRP, r^2^ = 0.9833; pThr849-TRP, r^2^ = 0.9911; pThr864-TRP, r^2^ = 0.9814) between the amount of TRP applied and the obtained Western blot signals (Fig. S2B). We then used these antibodies to verify the light-dependent phosphorylation of Thr849 and Thr864 observed in our quantitative mass spectrometric analysis (see Fig. 2) and to dissect the stages of the phototransduction cascade that are necessary to trigger phosphorylation of these sites. Wild type flies and mutants of the phototransduction cascade were illuminated for 12–18 h and then kept in the dark for 1 h or *vice versa*, before fly heads were subjected to Western blot analysis using α-pT849 and α-pT864 antibodies as well as an α-TRP antibody to monitor the total amount of TRP present in the sample (Fig. 3). In illuminated wild type flies, we observed strong phosphorylation of TRP at both Thr849 and Thr864 whereas in dark-adapted wild type flies, phosphorylation of Thr849 and Thr864 was barely detectable. These results are in accordance with the results obtained by mass spectrometry (see Fig. 2). The *ninaE^17^* mutant lacks the major rhodopsin of *Drosophila*, rhodopsin 1 [27], [33]. Phosphorylation of Thr849 and Thr864 in this mutant under both light conditions roughly resembled the phosphorylation levels observed in dark-adapted wild type flies. The *Gαq^1^* mutant harbors a hypomorphic mutation that leads to a thousand-fold reduction in light sensitivity [28]. As a result, light-adapted *Gαq^1^* mutants exhibited decreased phosphorylation of Thr849 and Thr864 as compared to light-adapted wild type flies. However, phosphorylation at both Thr849 and Thr864 was stronger in light-adapted *Gαq^1^* mutants as compared to dark-adapted *Gαq^1^* mutants. This latter finding may be explained by the residual photoreceptor activity that is due to the fact that *Gαq^1^* is not a null mutant. *norpA^P24^* mutant flies are blind because they lack phospholipase Cβ [29]. Phosphorylation of both Thr849 and Thr864 was reduced in the *norpA^P24^* mutant to a level resembling that of dark-adapted wild type flies. The *trp^D621G^* fly transgenically expresses a modified TRP channel which is impermeable for ions [31] in a *trp* null mutant background. In illuminated *trp^D621G^* flies, phosphorylation of Thr849 and Thr864 was drastically reduced as compared to illuminated wild type flies. Contrary to the *trp^D621G^* transgene, *trp^P365^* is a mutation that leads to a constitutively opened pore leading to permanent Ca^2+^ influx into the photoreceptor cells [26]. To rule out the possibility that the phosphospecific antibodies might not detect the *trp^P365^* gene product, and to attenuate degeneration caused by permanent Ca^2+^ influx, heterozygous 2-day-old flies (*trp^P365^*/+) were used. Phosphorylation of both Thr849 and Thr864 was considerably increased in dark-adapted *trp^P365^*/+ flies as compared to wild types. Taken together, we suggest that Ca^2+^ is the trigger for phosphorylation of both Thr849 and Thr864 because on the one hand Ca^2+^ influx into the photoreceptor cells is sufficient to evoke TRP phosphorylation but on the other hand a completely functional phototransduction cascade is not capable of triggering phosphorylation when TRP is impermeable for ions.

**Figure 4:**
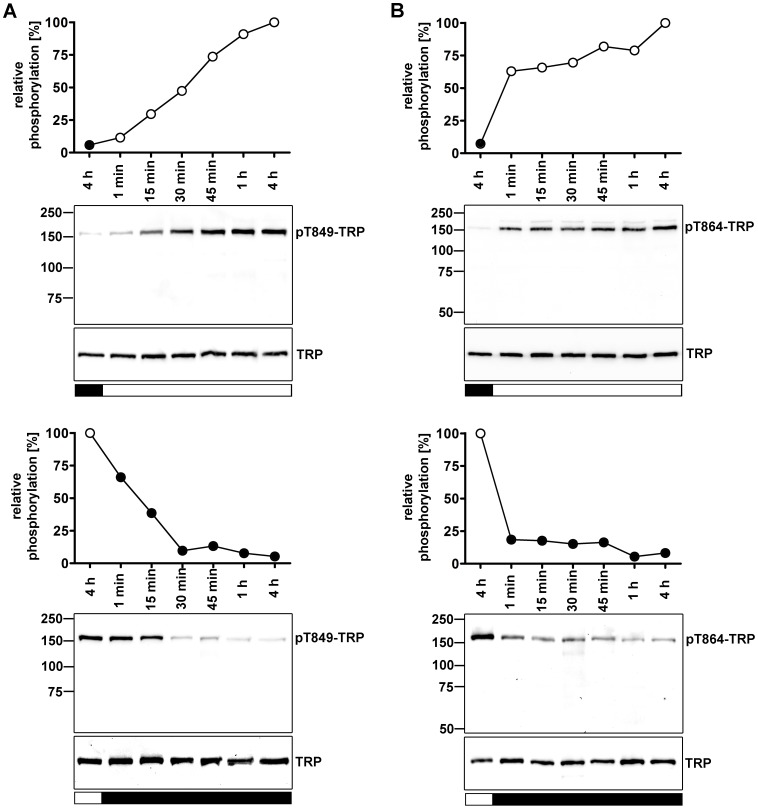
Time courses of Thr849 (A) and Thr864 (B) phosphorylation and dephosphorylation. Prior to protein extraction from fly heads, flies were kept in the dark or were illuminated for 12–18 h before they were subjected to the opposite light conditions for the indicated periods of time. Black bars indicate darkness and white bars indicate white light as the last light condition. Western blots were probed with α-pT849, α-pT864, and α-TRP antibodies as indicated. Signals obtained with the phosphospecific antibodies were normalized to signals obtained with α-TRP antibody. Phosphorylation levels after 4 h of light adaptation were set to 100%. Molecular mass markers (in kilodalton) are indicated to the left.

To investigate the time course of Thr849 and Thr864 phosphorylation and dephosphorylation, wild type flies were dark-adapted or illuminated for 12–18 h and were then subjected to the opposite light condition for different periods of time (Fig. 4). For both phosphorylation sites and regardless whether flies were switched from darkness to light or *vice versa*, already one min after changing the light conditions we detected significant changes in the phosphorylation state. However, phosphorylation and dephosphorylation kinetics were considerably faster for the Thr864 phosphorylation site as compared to Thr849.

### A Considerable Fraction of TRP is Phosphorylated at Thr849 and Thr864 in the Light

**Figure 5:**
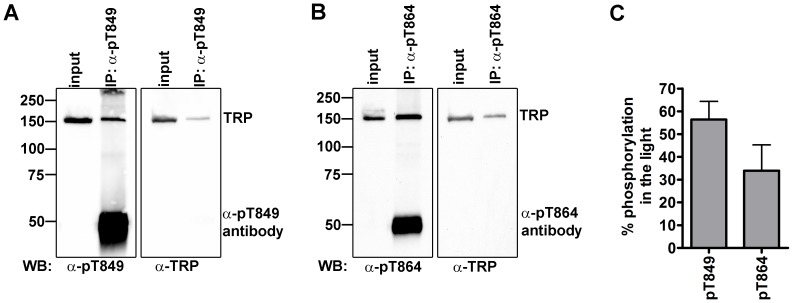
Phosphate occupancy of Thr849 and Thr864. Flies were kept in the dark for 12–18 h and were then illuminated for 1 h before proteins were extracted from heads. TRP phosphorylated at Thr849 and Thr864 was precipitated using α-pT849 (A) and α-pT864 (B) antibodies. Inputs and immunoprecipitates were loaded onto SDS gels and transferred to PVDF membranes. Membranes were probed with α-pT849 (A) and α-pT864 (B) and α-TRP antibodies as indicated. (C), Phosphate occupancies at Thr849 and Thr864 were calculated from three independent experiments by quantifying the Western blot signals of inputs and immunoprecipitations (see Materials and Methods) and using the formula OC = (α-pTRP_input_/α-TRP_input_) × (α-TRP_IP_/α-pTRP_IP_)×100% (see text for explanation). Error bars indicate SEM.

Our quantitative mass spectrometry and Western blot analyses yielded relative differences in phosphorylation between the light- and dark-adapted flies for each TRP phosphorylation site but did not provide information about the fraction of TRP molecules that are phosphorylated at a particular site relative to the total amount of TRP. We refer to this fraction as the phosphate occupancy of a phosphorylation site. To estimate the phosphate occupancy of Thr849 and Thr864, we employed the phospospecific antibodies directed against these sites and the generic α-TRP antibody that detected all TRP molecules. In order to be able to compare signals obtained with phosphospecific antibodies and the generic TRP antibody, TRP phosphorylated at Thr849 or Thr864 was immunoprecipitated with the respective phosphospecific antibody and the immunoprecipitates, which presumably contained phosphorylated TRP, were probed with both the corresponding phosphospecific antibody and the generic TRP antibody (Fig. 5A, B). Inputs that contained phosphorylated as well as non-phosphorylated TRP were probed with the same antibody combination on the same Western blot. The phosphate occupancy (OC) of Thr849 or Thr864 in percent was then calculated using the formula OC = (α-pTRP_input_/α-TRP_input_)×(α-TRP_IP_/α-pTRP_IP_)×100%, where α-pTRP_input_ and α-TRP_input_ are the signal intensities obtained with the phosphospecific and the generic TRP antibodies in the extracts before immunoprecipitation, and α-pTRP_IP_ and α-TRP_IP_ are the signal intensities from the immunoprecipitates. Assuming that all TRP molecules immunoprecipitated with the phosphospecific antibodies were phosphorylated, this analysis revealed that in the light, at most 56% (+/−13.8%) TRP molecules were phosphorylated at Thr849 and at most 34% (+/−19.6%) at Thr864 (Fig. 5C). However, note that TRP channels are immunoprecipitated as tetramers and not all members of a tetramer are necessarily phosphorylated. Co-precipitation of non-phosphorylated TRP would lead to an overestimation of phosphorylation. Even then, our analysis reveals that a considerable fraction of TRP becomes phosphorylated at Thr849 and Thr864 in the light.

### TRP Phosphorylated at Thr849 or Thr864 Resides in the Rhabdomeres

**Figure 6:**
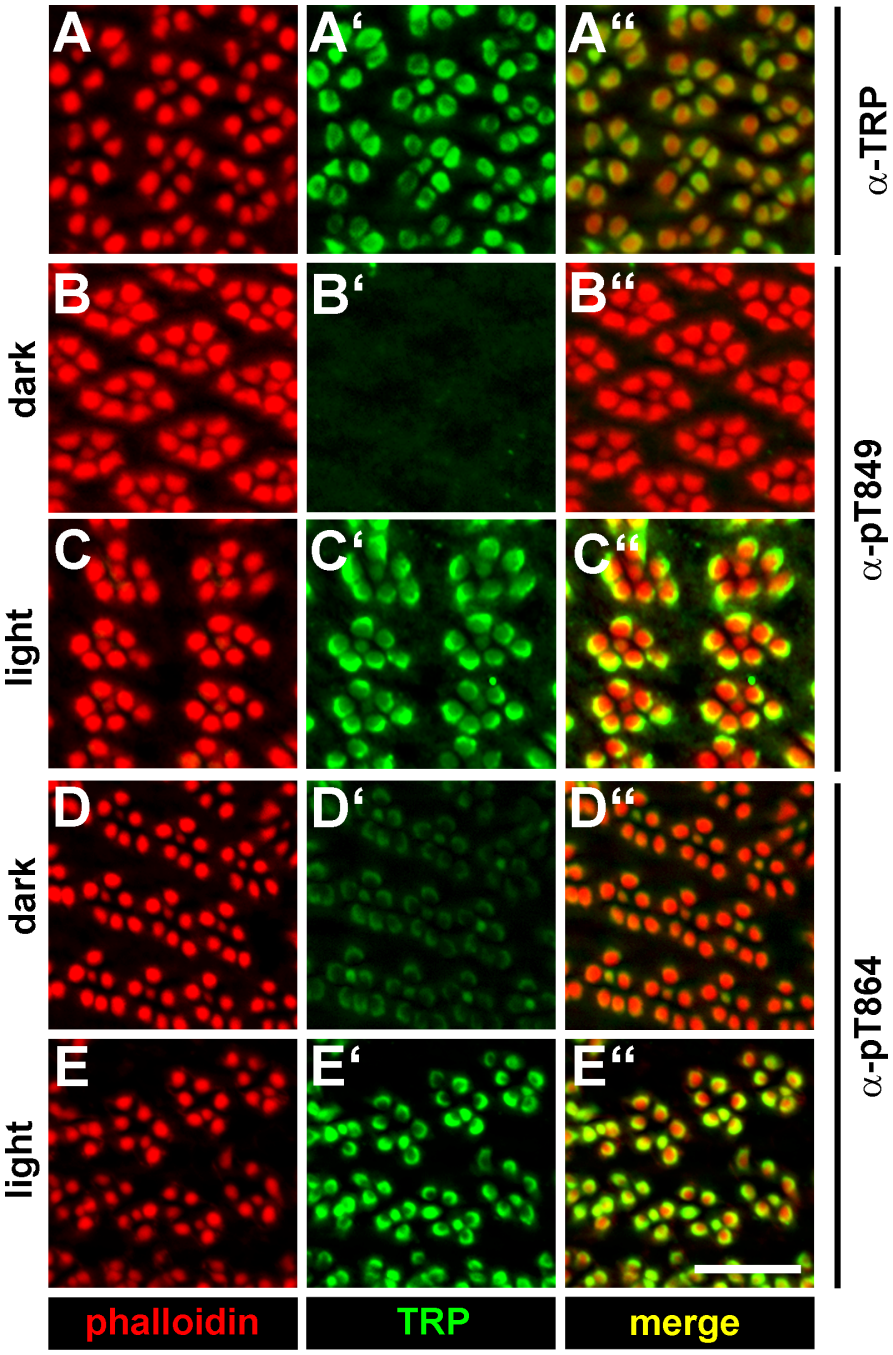
Immunocytochemistry of TRP phosphorylated at Thr849 and Thr864. Tangential cryosections through the eyes of wild type flies were probed with phalloidin staining the rhabdomeres (red), monoclonal α-TRP antibody (A–A′′), α-pT849 antibody (B–B′′, C–C′′), and α-pT864 antibody (D–D′′, E–E′′) (green). Overlay of signals from phalloidin and the respective antibody appears yellow in the merged panels. Scale bar, 10 µm.

Since phosphorylation might be a signal for subcellular targeting, we investigated the subcellular localization of TRP phosphorylated at Thr849 or Thr864 (Fig. 6). We found that both TRP phosphorylated at Thr849 and at Thr864 resided in the rhabdomeres of illuminated flies. However, the signals obtained with both phosphospecific antibodies were drastically diminished in dark-adapted flies. This finding further corroborates our results obtained by mass spectrometry in a recent publication [24] and in this study (see Fig. 2) and by Western blot analyses (Figs. 3 and 4). Our findings obtained by immunocytochemistry further suggest that phosphorylation of Thr849 and Thr864 takes place in the rhabdomere. Accordingly, the kinases and phosphatases mediating the phosphorylation and dephosphorylation of these two sites are expected to reside permanently or transiently in the rhabdomere.

### Candidate Screen to Identify TRP Kinases and Phosphatases

**Figure 7:**
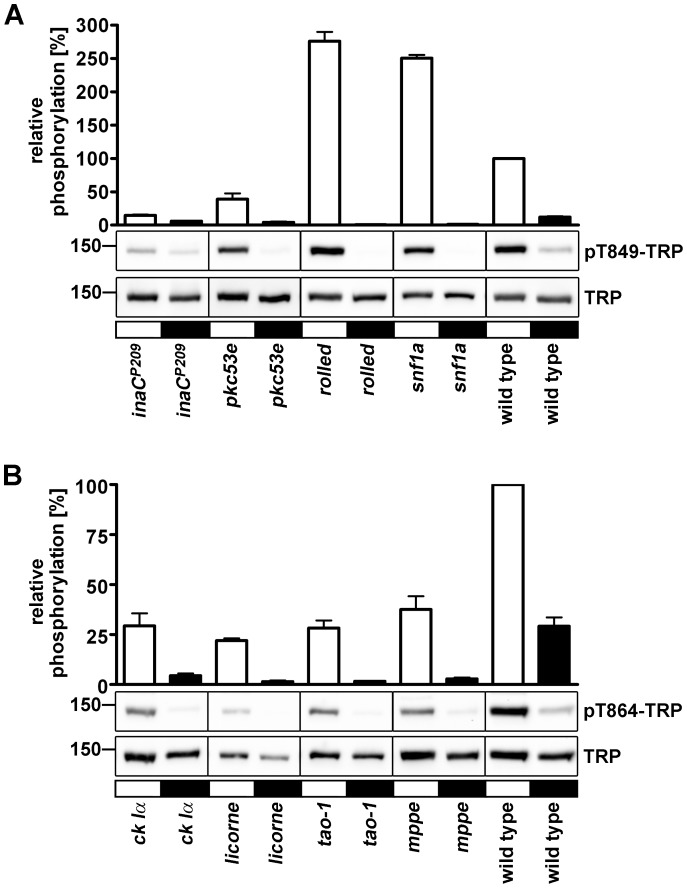
Selected results of a candidate screen of kinase and phosphatase mutants that affect TRP phosphorylation at Thr849 and Thr864. Flies were illuminated for 12–18 h and then kept in the dark for 1 h (black bars) or *vice versa* (white bars) before they were subjected to Western blot analysis using α-pT849 and α-pT864 antibodies. All Western blots were reprobed with monoclonal α-TRP antibody to reveal the amount of TRP present in the samples. The lower panels show representative Western blots using α-pT849, α-pT864, and α-TRP antibodies. Molecular mass markers (in kilodalton) are indicated to the left. The diagram at the top shows a quantification of signals obtained with the phosphospecific antibodies from 3 independent experiments for each mutant that were normalized to the signals obtained with the generic α-TRP antibody. Relative Phosphorylation in percent was obtained by comparing the values of the mutants with the values of light-adapted wild type flies (set to 100%) on the same Western blot. Error bars show SEM (n = 3). All results of the screen are depicted in Table S2. Vertical black lines indicate that the signals were taken from different membranes.

In order to identify kinases and phosphatases of Thr849 and Thr864 of the TRP ion channel, we conducted a candidate screen using the two phosphospecific antibodies α-pT849 and α-pT864 (Fig. 7). We screened 85 *Drosophila* mutants with defects in kinases and phosphatases that are expressed in the eye. A list of the used mutants and the detailed results of the screen are provided in table S2. Flies were light- or dark-adapted for 12–18 h and were then switched to the opposite light condition for 1 h before fly heads were subjected to Western blot analysis. Flies that showed an up-regulation of at least 2fold or down-regulation of less than 0.5fold in TRP phosphorylation at Thr^849^ or Thr^864^ as compared to wild type controls in one light condition (light or dark) in this initial screen were tested again two more times. Out of the 85 tested mutants, eight displayed altered phosphorylation of Thr849 and Thr864. The rescreen revealed significant effects on phosphorylation in four mutants in case of Thr849 and four mutants in case of Thr864 (Fig. 7).

Thr849 phosphorylation was reduced in light-adapted *inaC^P209^* mutants that lack eye-PKC that has been shown to phosphorylate TRP and INAD *in vitro* [6], [7]. The other canonical PKC in *Drosophila* is PKC53E. The *pkc53E* mutant also exhibited a decrease in Thr849 phosphorylation in the light. In contrast, flies with impairments in *rolled* and *snf1a* showed increased phosphorylation of Thr849 in the light. Thr864 phosphorylation was reduced in light-adapted flies with impairments in *ckI alpha*, *licorne*, *tao-1*, and *mppe* genes.

Since we concluded that a proper subcellular localization of TRP in the rhabdomere is essential for its accessibility by kinases and phosphatases, we analyzed the subcellular localization of TRP in the fly strains that displayed an altered phosphorylation pattern of Thr849 and Thr864 (Fig. S3). As a result, none of the flies exhibited mislocalized TRP ruling out the possibility that altered phosphorylation resulted from mislocalization of TRP. To rule out an effect of visual impairment, we also recorded ERGs from flies showing altered phosphorylation of TRP. As a result, all flies with altered phosphorylation at T849 and T864 exhibited ERGs with normal amplitudes (data not shown).

## Discussion

We previously identified 21 phosphorylation sites in the *Drosophila* TRP channel and showed that a subset of these sites is phosphorylated light-dependently [24]. In the current study, we identified seven novel phosphorylation sites in the C-terminal region of TRP (Ser867, Ser958, Thr998, Thr1036, Thr1049, Ser1123, and Ser1253). We extended the previous results by showing that the light-triggered phosphorylation of 13 sites (Ser726, Thr849, Thr864, Ser872, Ser876, Ser958, Ser961, Ser964, Ser982, Thr998, Ser1056, Ser1123, and Ser1254) as well as the dephosphorylation of Ser936 depends on the presence of NORPA and hence on activation of the phototransduction cascade. We further provide evidence that the phosphorylation of at least seven of these sites (Thr849, Thr864, Ser872, Ser876, Ser961, Ser982, and Ser1056) and also the light-dependent dephosphorylation of Ser936 are triggered by a constitutively active TRP channel in *trp^P365^*/+ flies possibly due to light-independent Ca^2+^ influx into photoreceptor cells. In the dark, the phototransduction cascade is not activated but in the *trp^P365^*/+ flies there is a sustained Ca^2+^ influx into the photoreceptor cells due to the constitutive activity of the *trp^P365^* gene product. This resulted in a TRP phosphorylation pattern that was significantly different from the pattern observed in dark-adapted wild type flies. Because the TRP phosphorylation sites are embedded within consensus sequences for different kinases, this result is rather unexpected. The most obvious explanation for this finding would be that most of the kinases that mediate light-dependent TRP phosphorylation and the phosphatases that mediate light-dependent TRP dephosphorylation are directly or indirectly regulated by Ca^2+^.

Our detailed analysis of the two light-dependent TRP phosphorylation sites, Thr849 and Thr864, using phosphospecific antibodies provided further insight into the regulation of TRP phosphorylation and identified involved kinases and phosphatases. Thr849 and Thr864 were found to have a high level of phosphate occupancy in the light. At most 56% of all TRP molecules were phosphorylated at Thr849 and at most 34% were phosphorylated at Thr864. The phosphate occupancy was calculated using the two phosphospecific and a generic TRP antibody. In order to correct for differences in antibody reactivity, we compared the Western blot signal intensities of TRP that was immunoprecipitated with the phosphospecific antibodies. This quantification relies on the assumption that only phosphorylated TRP is precipitated by the phosphospecific antibodies. We tested this assumption by immunoprecipitating TRP isolated from dark-adapted flies that show little phosphorylation at Thr849 and Thr864 with phosphospecific antibodies (data not shown). As expected, the amount of TRP detected with the generic TRP antibody was much lower (about 7fold lower for α-Thr849 as well as α-Thr864) in these immunoprecipitates as compared to immunoprecipitates obtained from light-adapted flies. This difference in the amount of immunoprecipitated TRP correlates well with the difference in phosphorylation at Thr849 and Thr864 that was determined by quantitative Western blot analysis to be 5.4fold higher for Thr849 and 3.9fold higher for Thr864 in the light. It is important to note that TRP is likely immunoprecipitated as tetramers. Thus, using phosphospecific antibodies to pull down phosphorylated TRP may result in coprecipitation of non-phosphorylated TRP. This would lead to an overestimation of phosphate occupancy. Additionally, some uncertainty remains with respect to the signal of the two phosphospecific antibodies on Western blots with extracts from dark-adapted flies. These signals could result from residual phosphorylation at Thr849 and Thr864 in the dark or from cross-reactivity of the phosphospecific antibodies with non-phosphorylated TRP. In the latter case, the phosphate occupancy of Thr849 and Thr864 would be slightly overestimated. Even then, since TRP channels are tetramers and phosphorylation of one subunit may already affect channel function, these phosphorylations could potentially regulate all TRP channels present in the photoreceptor.

As suggested by the light-independent phosphorylation of TRP sites in the *trp^P365^*/+ mutant, elevated intracellular Ca^2+^ levels might enhance phosphorylation of Thr849 and Thr864. However, phosphorylation of Thr849 was stronger in light- than in dark-adapted *trp^P365^*/+ flies (see Fig. 3A). This observation might be explained with the dependence of Thr849 phosphorylation on eye-PKC and PKC53E as revealed by our screen for TRP kinases and phosphatases. In addition to Ca^2+^, eye-PKC and PKC53E are synergistically activated by diacyl glycerol which is only generated when the phototransduction cascade is activated. Thus, while these protein kinases C presumably are fully activated in light-adapted *trp^P365^*/+ flies, they may only be partially activated in dark-adapted *trp^P365^*/+ flies. By contrast, Thr864 phosphorylation was not compromised in the *inaC^P209^* mutant and did not exhibit light-dependent changes in its phosphorylation state in the *trp^P365^*/+ fly (see Fig. 3B). In line with the hypothesis that elevated intracellular Ca^2+^ levels triggered phosphorylation of Thr849 and Thr864, the disruption of the TRP pore in the *trp^D621G^* fly abolished light-dependent phosphorylation of these two sites. The time course of Thr864 phosphorylation and dephosphorylation was faster than that of Thr849. However, already one minute after the switch of light conditions was a change in the phosphorylation states of both sites detectable. Because TRP phosphorylated at Thr849 and Thr864 resided exclusively in the rhabdomeres, we conclude that this is the place where phosphorylation and dephosphorylation of these sites take place. Therefore, it is not likely that phosphorylation of TRP at Thr849 and Thr864 is a signal for TRP translocation outside of the rhabdomeres. Instead, the rhabdomeric localization of TRP phosphorylated at Thr849 and Thr864 and the fast time course of phosphorylation might point to an involvement in regulation of the photoresponse. However, since kinase and phosphatase mutants with altered phosphorylation levels at Thr849 and Thr864 displayed normal ERG amplitudes, a thorough investigation of other ERG parameters such as response inactivation and deactivation time would be needed to further explore this possibility.

Our candidate screen for kinases and phosphatases revealed that phosphorylation of Thr849 was significantly reduced in the eye-PKC mutant *inaC^P209^*. Eye-PKC is a member of the INAD signaling complex and was shown to phosphorylate TRP *in vitro* [6], [7]. We found that phosphorylation of Thr849 was reduced 6.7 fold in the eye-PKC null mutant *inaC^P209^* while phosphorylation of Thr864 was unaltered. These findings are in accordance with the prediction that Thr849 is embedded within a PKC consensus sequence (according to the NetPhosK prediction algorithm). We therefore conclude that phosphorylation of Thr849 not only depends on the phototransduction cascade and Ca^2+^ but also on eye-PKC. The current finding that TRP phosphorylation at Thr849 depends on eye-PKC seems to conflict with our previous finding that TRP was still light-dependently phosphorylated in the eye-PKC null mutant [24]. However, in our current study we also observed considerable light-dependent phosphorylation at this site in the eye-PKC null mutant albeit at a much lower level than in wild-type flies. We conclude that another kinase acts redundantly in phosphorylating this TRP phosphorylation site. Besides eye-PKC, which belongs to the subgroup of canonical PKCs with homology to vertebrate PKCα and PKCβ, only one other canonical PKC has been identified in *Drosophila*, PKC53E [34], [35]. The genes encoding eye-PKC and PKC53E are located in close proximity at position 53E of the second chromosome, and it has been suggested that they derived from a gene duplication event [30]. Both eye-PKC and PKC53E are expressed in the retina while PKC53E is also expressed in the brain [35]. PKC53E therefore is an obvious candidate for a kinase that might substitute or act together with eye-PKC in phosphorylation of TRP. Indeed, we found that the *pkc53e* null mutant exhibited reduced phosphorylation of TRP at Thr849. We therefore conclude that *in vivo*, eye-PKC and PKC53E act redundantly in phosphorylating TRP at Thr849 and possibly at other sites that are embedded within PKC consensus sequences. Non-canonical PKCs that were tested in our screen (PKC98E, PKN, and DaPKC) were not found to affect phosphorylation at Thr849. Enhanced phosphorylation at Thr849 in the light was observed in two of the tested mutants, *rolled* and *snf1a.* The rolled (*rl*) gene encodes a mitogen-activated protein kinase [36] that is involved in many biological processes including eye development [37], [38]. We therefore cannot rule out a secondary effect in the *rolled* mutant on TRP phosphorylation. The *rl^1^* allele used for the screen is a hypomorphic loss-of-function allele. The *snf1a* gene encodes an AMP-activated protein kinase that has been implicated in the cellular energy sensing pathway [39].

The TRP phosphorylation site Thr864 showed reduced phosphorylation in light-adapted flies in four of the tested mutants, *casein kinase 1α*, *licorne*, *tao-1* and *mppe*. However, in neither of these mutants light-dependent phosphorylation of Thr864 was completely abolished, ruling out the possibility that one of these kinases is uniquely responsible for phosphorylation of this site. *Drosophila* casein kinase 1α (CK1α) is a negative regulator of the wingless (Wg) pathway. It phosphorylates the Armadillo protein and thereby stimulates its degradation [40]. Among other tissues, CK1α is highly expressed in the adult eye (flyatlas). The *ck1α* mutant we used in our screen harbors a transposable element disrupting the *ck1α* gene. Licorne is a MAP kinase kinase [41] that is moderately expressed in all adult tissues (www.flybase.org). TAO-1 is a protein serine/threonine kinase of the STE family that controls microtubule dynamics, animal behavior, brain development, and epithelial tissue growth [42]–[45]. According to flyatlas, in adult flies, *tao-1* is strongly expressed in the eye and ovary. The *tao-1* mutant used in our screen harbors a transposable element in the 5′ region of the *tao-1* gene.

MPPE is a Mn^2+^/Zn^2+^-dependent metallophosphoesterase that desphosphorylates and thereby activates α-Man-II [46]. The latter is needed to deglycosylate Rh1 during its maturation. The *mppe* mutant fly exhibits incomplete deglycosylation of Rh1. Subcellular localization and signaling of Rh1 are not affected in this mutant, but incomplete deglycolsylation causes morphological and functional defects in aged flies. In our screen, the *mppe* mutant exhibited a reduced phosphorylation of Thr864. Since MPPE is a protein phosphatase, loss of MPPE activity should result in a higher degree of phosphorylation if MPPE would directly dephosphorylate the respective site. We therefore conclude that MPPE is indirectly involved in Thr864 phosphorylation, for example by activating a kinase through dephosphorylation or by affecting the glycosylation state of proteins involved in TRP phosphorylation.

Our data suggest that regulation of most TRP phosphorylation sites depends on the phototransduction cascade. Although the TRP phosphorylation pattern is shaped by different kinases and phosphatases, most of them seem to be regulated by Ca^2+^.

## Supporting Information

Figure S1 **Representative MS/MS spectra of newly identified TRP phosphorylation sites.** Figures S1A, S1C, S1D, S1E, S1F, and S1G show fragmentation spectra and tables derived from tryptic TRP peptides and figure S1B shows a fragmentation spectrum derived from a chymotryptic TRP peptide. The coverage of the peptide sequence by b- and y-ions, and the calculated mass for each fragment ion are shown in the tables below the spectra, in which observed b- and y-ions are highlighted in red and blue, respectively.
(DOCX)

Figure S2 **Validation of antibodies.** A, To check for cross-reactivity with other phosphorylation sites or the non-phosphorylated peptide, 2.5 µg of phospho- and dephosphopeptides were applied onto nitrocellulose membranes and the membranes were then blocked and incubated with α-pT849 or α-pT864 antibodies and a secondary α-rabbit IgG conjugated to horse radish peroxidase. Enhanced chemiluminescence signals were recorded. Phosphopeptides were NH_2_-CGRKK(pT)QKGD-CONH_2_ containing phosphorylated Thr849 of TRP, NH_2_-CARKN(pT)FASD-CONH_2_ containing phosphorylated Thr864 of TRP, and NH_2_-CADEVpSLADD-CONH_2_ containing phosphorylated Ser936 of TRP. The dephosphopeptides were similar to the respective phosphopeptides except for the lack of the phosphoryl groups. B, To check linearity of the signal intensities obtained with the antibodies, different amounts of protein extracts from wild type heads were supplemented with protein extracts from *trp^P343^* null mutant heads to ensure equal overall protein content. Three head equivalents were loaded onto a gel and subjected to Western blot analysis using α-TRP antibody. After stripping, either α-pT849 or α-pT864 antibodies were used. These experimental conditions correspond to those used in the screen to identify kinases or phosphatases of TRP at T849 and T864. Error bars show SEM (N = 4).
(TIF)

Figure S3 **Immunostaining of isolated ommatidia to investigate the subcellular localization of TRP in mutants used in the screen.** Immunostaining of isolated ommatidia was carried out essentially as described (20). Briefly, flies were decapitated and heads were cut into halves. Eyes were dissected in 100 mM phosphate buffer, pH 7.2, using forceps. Fragments were pipetted through a 200 µl pipette tip several times and transferred to a lysine-coated cover slide and air-dried. Fixation was accomplished by a 10 min incubation with 2% paraformaldehyde in 1×PBS (130 mM NaCl, 7 mM Na_2_HPO_4_, 1 mM NaH_2_PO_4_), pH 7.2, followed by a two 5 min washes with 1×PBS-S (PBS containing 0.1% (w/v) saponine). Ommatidia were permeabilized by incubation in cytoskeleton buffer (200 mM sucrose, 10 mM Hepes, pH 7.4, 3 mM MgCl_2_, 50 mM NaCl, 0.5% Triton X-100, 0.02% NaN_3_) for 8 min at RT and washed three times with PBS-S for five min each. Incubation in primary antibody (α-TRP) was carried out in blocking solution (1×PBS containing 0.5% fish gelatine and 0.1% ovalbumine) over night at 4°C. Ommatidia were washed three times with PBS-S and then incubated for 2–4 h in secondary antibody (α-mouse Cy5 (Dianova)) and AlexaFluor 546-coupled phalloidin (Invitrogen) in blocking solution at ambient temperature followed by a three-times-wash with aqua bidest. Ommatidia were mounted with Mowiol 4.88 and were examined with an AxioImager.Z1m microscope (objective EC Plan-Neofluar x40/1.3 oil, Zeiss) with the ApoTome module (Zeiss) and recorded with an AxioCam MRm (Zeiss). Scale bar, 20 µm.
(TIF)

Table S1 **Peptides used for quantification of TRP phosphorylation sites.** Phosphorylated amino acids are underscored. p-values were calculated by an unpaired t-test using the normalized abundances from the two conditions given in each table. SD, standard deviation.
(DOCX)

Table S2 **Listing of flies used for the candidate screen to identify kinases and phosphatases of TRP and detailed results.**
(DOCX)
