# Supplementary material for: Phosphorylation of the Drosophila Transient Receptor Potential Ion Channel Is Regulated by the Phototransduction Cascade and Involves Several Protein Kinases and Phosphatases
Source: PLoS One. 2013 Sep 9;8(9):e73787. doi: 10.1371/journal.pone.0073787 (PMC3767779; doi:10.1371/journal.pone.0073787)

**A**

pT864, pS867

KNpTFApSDPIGSK

m/z = 712.7965 (+2)


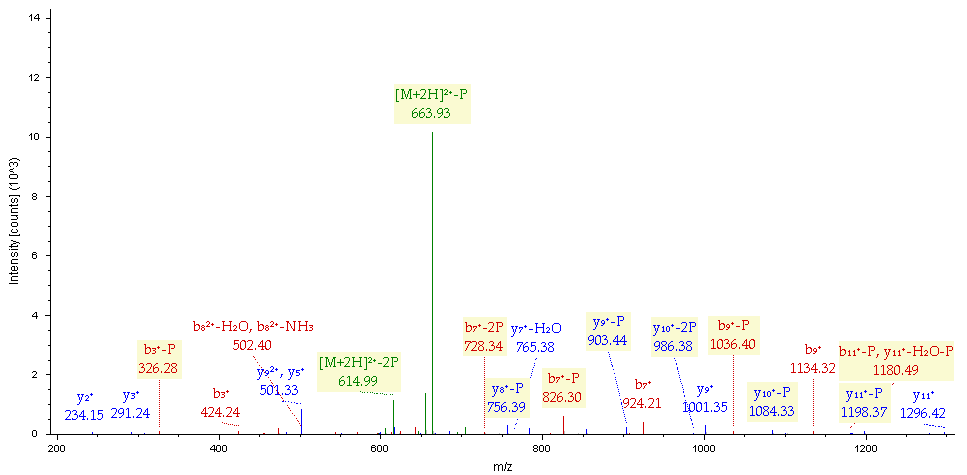


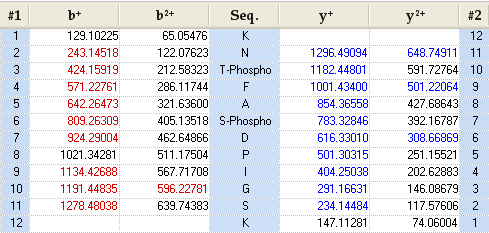


**B**

pS958

DASGpSKKSITSGGTGGGASoxML

m/z = 655.6261 (+3)


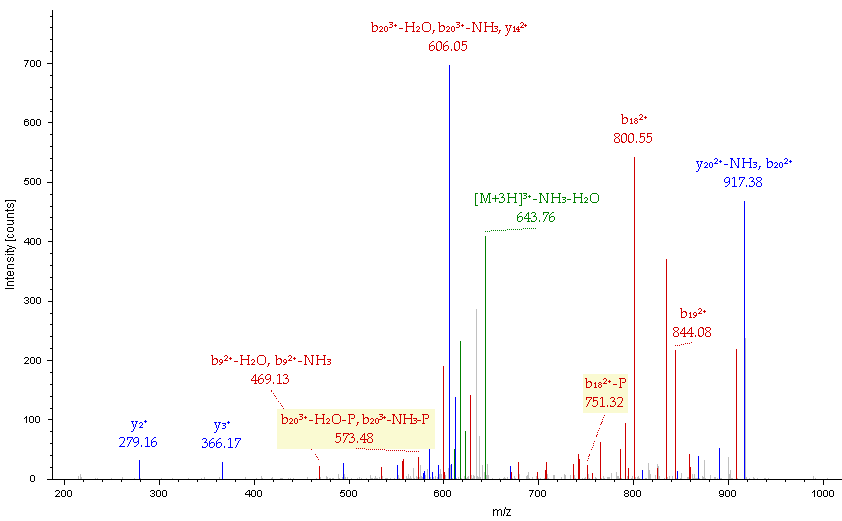


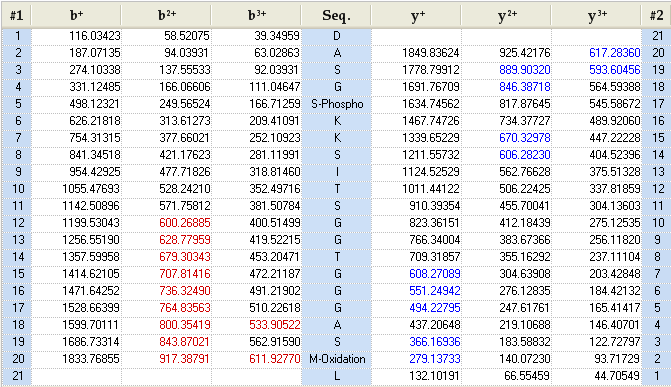


**C**

pT998

SGADGKPGpTMGKPTDDK

m/z = 581.2536 (+3)


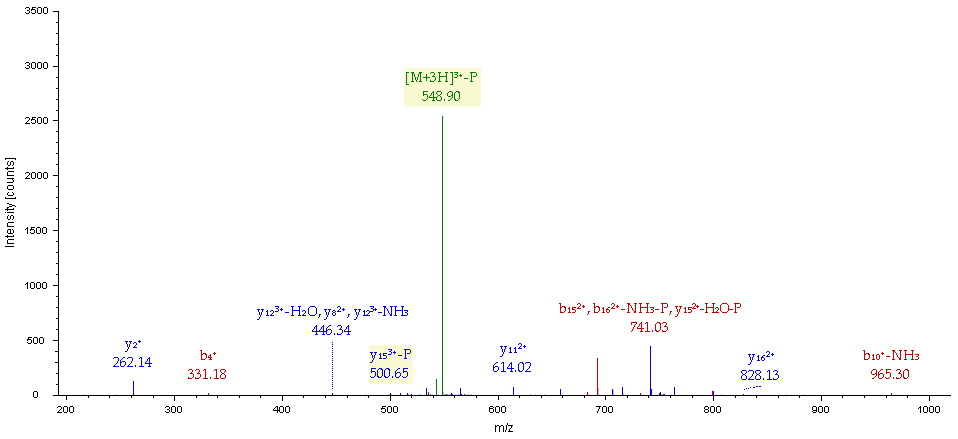


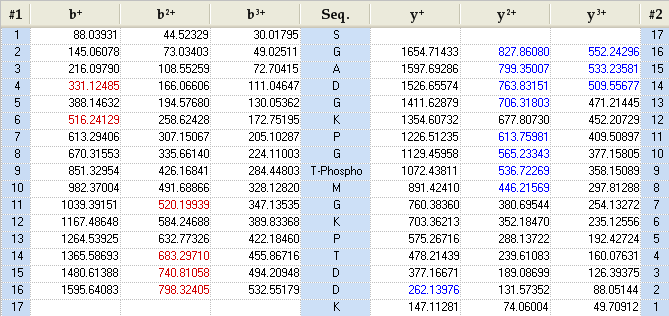


**D**

pT1036

DSKPSAGGPKPGDQKPpTPGAGAPK

m/z = 582.0363 (+4)


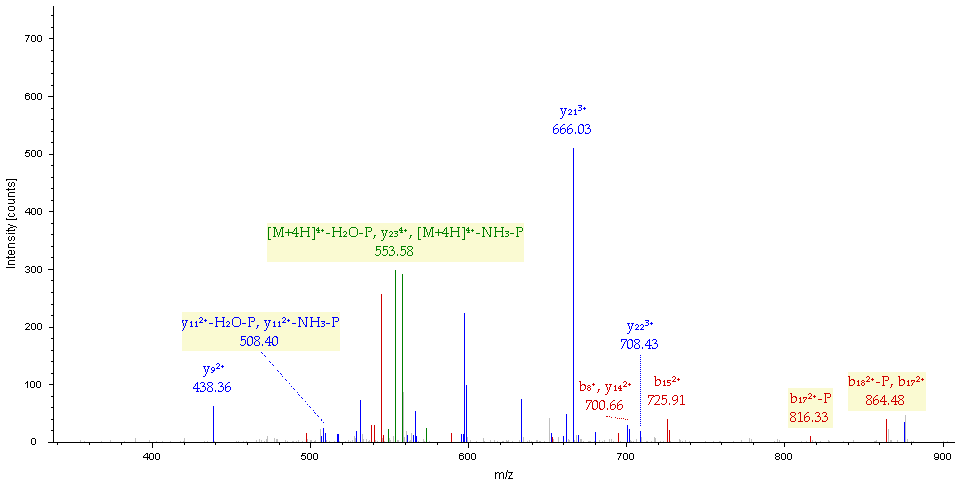


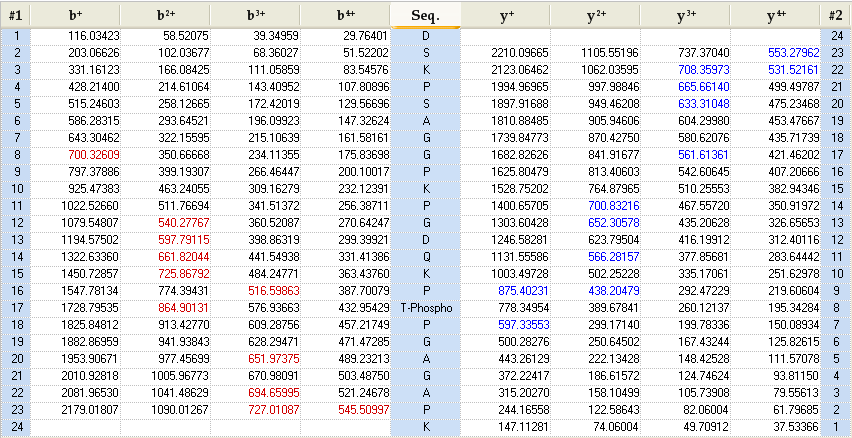


**E**

pT1049

PQAAGpTISKPGESQK

m/z = 526.9224 (+3)


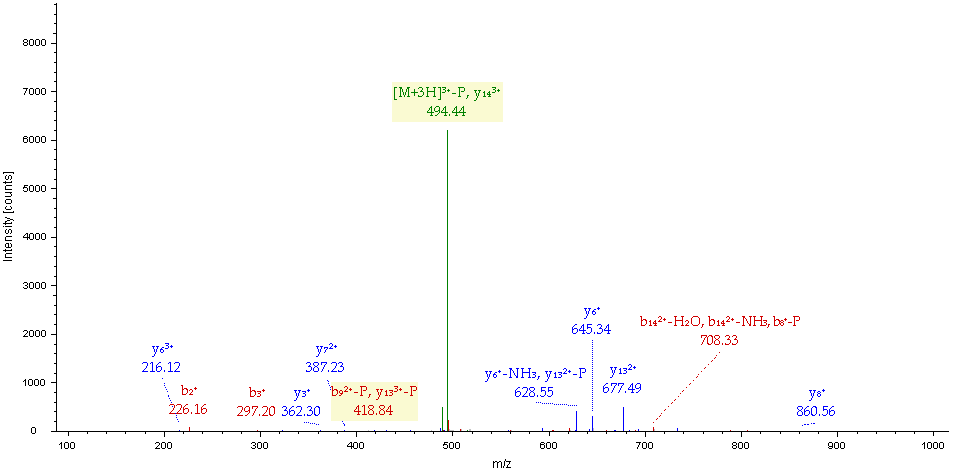


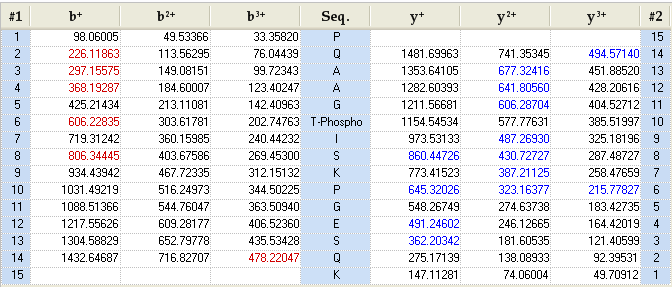


**F**

pS1123

SAAPSAPSDAKPDpSK

m/z = 503.5591 (+3)


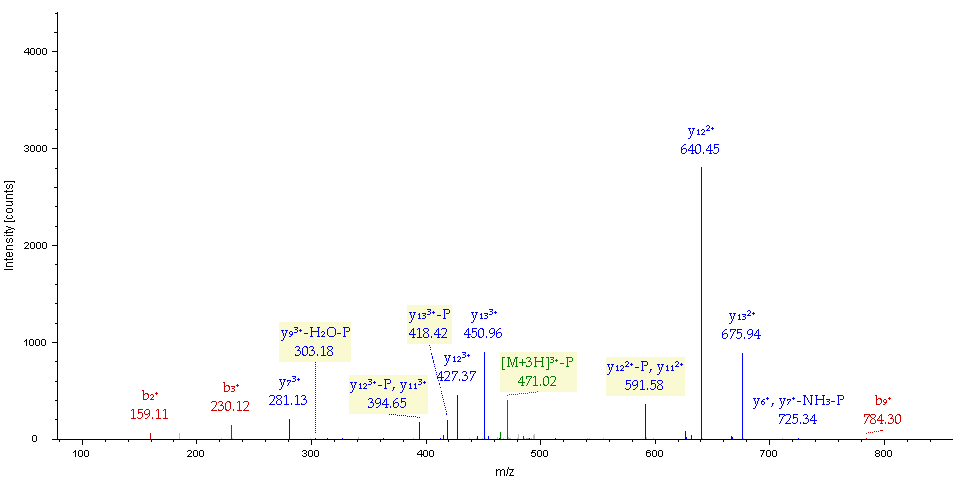


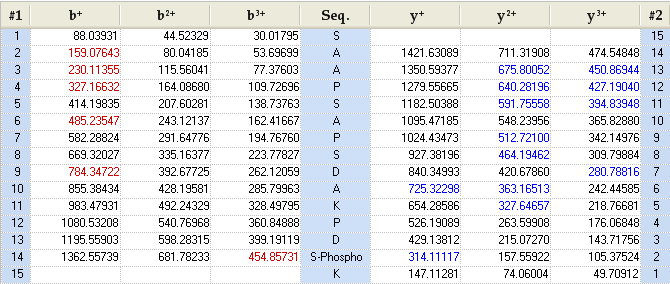


**G**

pS1253

VGQpSSAAAGGER

m/z = 585.2509 (+2)


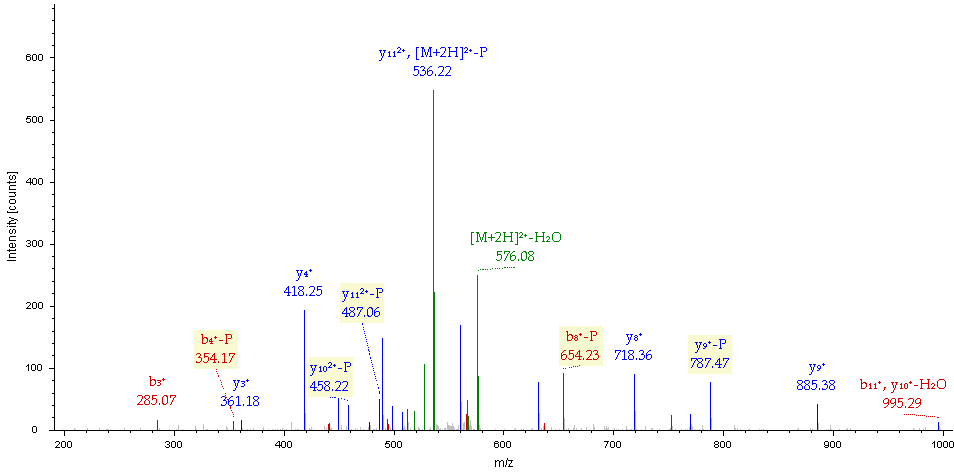


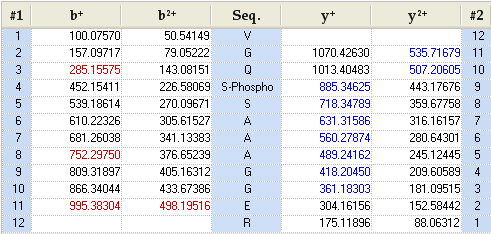

Supplement: Figure S1 — Representative MS/MS spectra of newly identified TRP phosphorylation sites. Figures S1A, S1C, S1D, S1E, S1F, and S1G show fragmentation spectra and tables derived from tryptic TRP peptides and figure S1B shows a fragmentation spectrum derived from a chymotryptic TRP peptide. The coverage of the peptide sequence by b- and y-ions, and the calculated mass for each fragment ion are shown in the tables below the spectra, in which observed b- and y-ions are highlighted in red and blue, respectively. (DOCX) [file pone.0073787.s001.docx]
