# Supplementary figures and images for: Phosphorylation of the Drosophila Transient Receptor Potential Ion Channel Is Regulated by the Phototransduction Cascade and Involves Several Protein Kinases and Phosphatases
Source: PLoS One. 2013 Sep 9;8(9):e73787. doi: 10.1371/journal.pone.0073787 (PMC3767779; doi:10.1371/journal.pone.0073787)

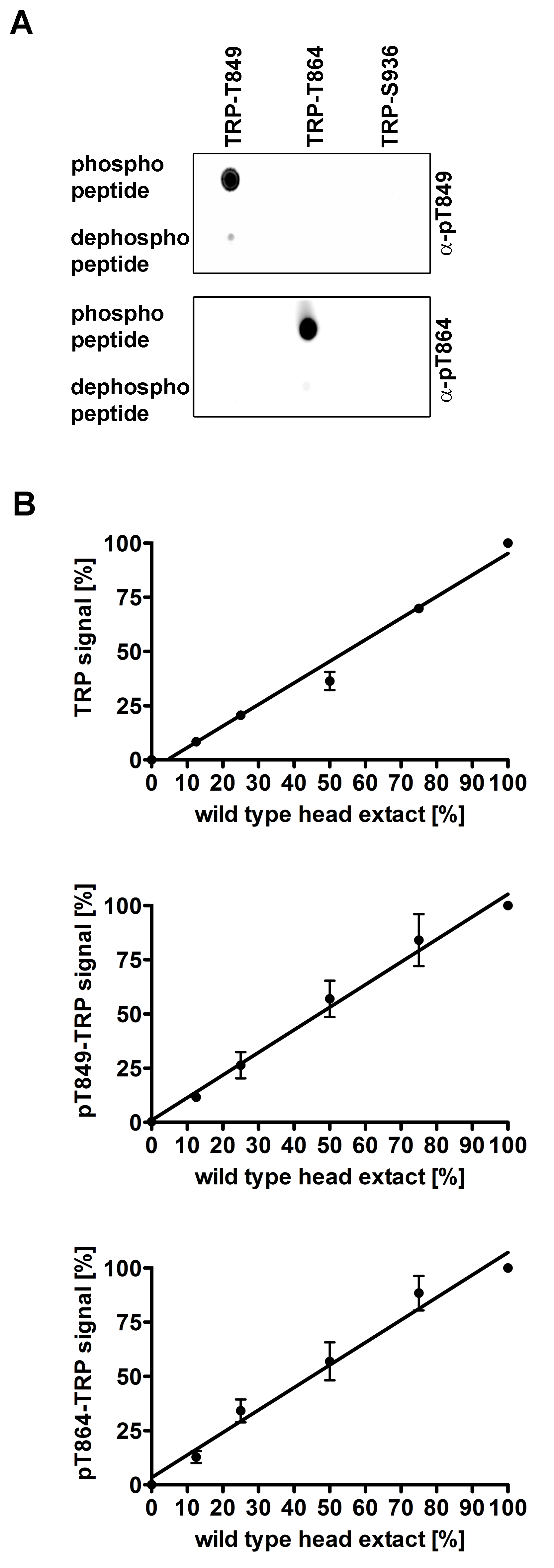

Supplement: Figure S2 — Validation of antibodies. A, To check for cross-reactivity with other phosphorylation sites or the non-phosphorylated peptide, 2.5 µg of phospho- and dephosphopeptides were applied onto nitrocellulose membranes and the membranes were then blocked and incubated with α-pT849 or α-pT864 antibodies and a secondary α-rabbit IgG conjugated to horse radish peroxidase. Enhanced chemiluminescence signals were recorded. Phosphopeptides were NH2-CGRKK(pT)QKGD-CONH2 containing phosphorylated Thr849 of TRP, NH2-CARKN(pT)FASD-CONH2 containing phosphorylated Thr864 of TRP, and NH2-CADEVpSLADD-CONH2 containing phosphorylated Ser936 of TRP. The dephosphopeptides were similar to the respective phosphopeptides except for the lack of the phosphoryl groups. B, To check linearity of the signal intensities obtained with the antibodies, different amounts of protein extracts from wild type heads were supplemented with protein extracts from trpP343 null mutant heads to ensure equal overall protein content. Three head equivalents were loaded onto a gel and subjected to Western blot analysis using α-TRP antibody. After stripping, either α-pT849 or α-pT864 antibodies were used. These experimental conditions correspond to those used in the screen to identify kinases or phosphatases of TRP at T849 and T864. Error bars show SEM (N = 4). (TIF) [file pone.0073787.s002.tif]

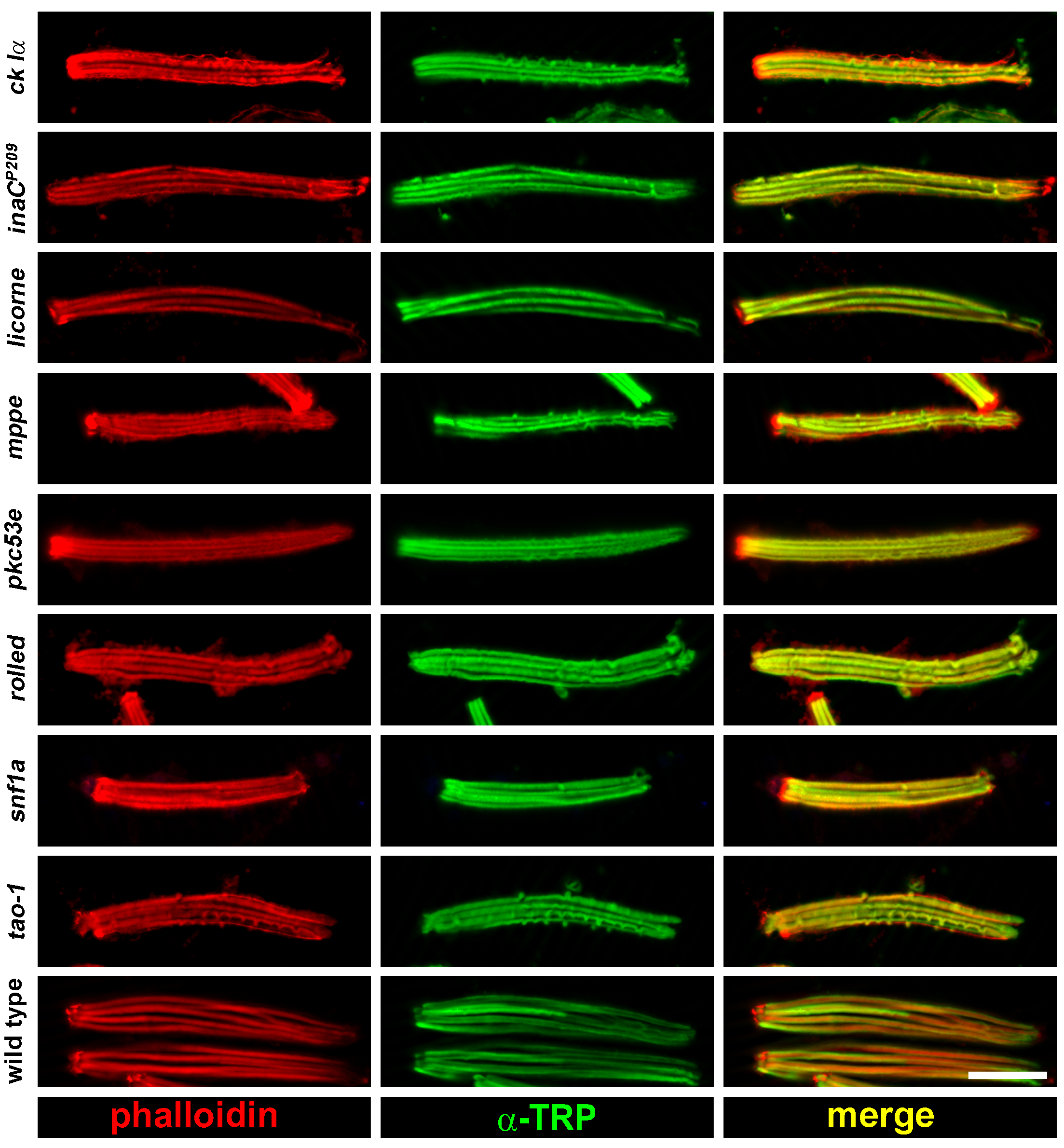

Supplement: Figure S3 — Immunostaining of isolated ommatidia to investigate the subcellular localization of TRP in mutants used in the screen. Immunostaining of isolated ommatidia was carried out essentially as described (20). Briefly, flies were decapitated and heads were cut into halves. Eyes were dissected in 100 mM phosphate buffer, pH 7.2, using forceps. Fragments were pipetted through a 200 µl pipette tip several times and transferred to a lysine-coated cover slide and air-dried. Fixation was accomplished by a 10 min incubation with 2% paraformaldehyde in 1×PBS (130 mM NaCl, 7 mM Na2HPO4, 1 mM NaH2PO4), pH 7.2, followed by a two 5 min washes with 1×PBS-S (PBS containing 0.1% (w/v) saponine). Ommatidia were permeabilized by incubation in cytoskeleton buffer (200 mM sucrose, 10 mM Hepes, pH 7.4, 3 mM MgCl2, 50 mM NaCl, 0.5% Triton X-100, 0.02% NaN3) for 8 min at RT and washed three times with PBS-S for five min each. Incubation in primary antibody (α-TRP) was carried out in blocking solution (1×PBS containing 0.5% fish gelatine and 0.1% ovalbumine) over night at 4°C. Ommatidia were washed three times with PBS-S and then incubated for 2–4 h in secondary antibody (α-mouse Cy5 (Dianova)) and AlexaFluor 546-coupled phalloidin (Invitrogen) in blocking solution at ambient temperature followed by a three-times-wash with aqua bidest. Ommatidia were mounted with Mowiol 4.88 and were examined with an AxioImager.Z1m microscope (objective EC Plan-Neofluar x40/1.3 oil, Zeiss) with the ApoTome module (Zeiss) and recorded with an AxioCam MRm (Zeiss). Scale bar, 20 µm. (TIF) [file pone.0073787.s003.tif]
