# Supplementary material for: Phosphorylation of the Drosophila Transient Receptor Potential Ion Channel Is Regulated by the Phototransduction Cascade and Involves Several Protein Kinases and Phosphatases
Source: PLoS One. 2013 Sep 9;8(9):e73787. doi: 10.1371/journal.pone.0073787 (PMC3767779; doi:10.1371/journal.pone.0073787)
Supplement: Table S1 — Peptides used for quantification of TRP phosphorylation sites. Phosphorylated amino acids are underscored. p-values were calculated by an unpaired t-test using the normalized abundances from the two conditions given in each table. SD, standard deviation. (DOCX) [file pone.0073787.s004.docx]

**Table S1A. Tryptic peptides for quantification of wild type light versus wild type dark.**

| **phosphorylation site** | **peptide** | **m/z** | **charge** | **mean normalized abundance (wild type light)** | **SD (wild type light)** | **mean normalized abundance (wild type dark)** | **SD (wild type dark)** | **p-value** |
| --- | --- | --- | --- | --- | --- | --- | --- | --- |
| S721 | TKSFMR | 425.1882 | 2 | 88.31 | 49.84 | 41.45 | 34.57 | 3.400E-02 |
| T849 | KKTQKGDKDWNAIAR | 460.4873 | 4 | 24009.80 | 8153.99 | 92.68 | 46.31 | 1.000E-04 |
| T864 | KNTFASDPIGSK | 672.8133 | 2 | 40113.11 | 20182.35 | 555.37 | 144.35 | 1.000E-04 |
| S872 | KNTFASDPIGSKR | 500.9117 | 3 | 40036.01 | 28273.44 | 34.22 | 27.17 | 6.000E-04 |
| S936 | M(ox)AADEVSLADDEGAPNGEGEK | 1100.9334 | 2 | 4.25 | 12.75 | 11638.81 | 9498.77 | 2.000E-03 |
| S961 | SITSGGTGGGASM(ox)LAAAALR | 615.6239 | 3 | 1645.93 | 1204.03 | 21.29 | 34.09 | 9.000E-04 |
| S964 | SITSGGTGGGASM(ox)LAAAALR | 922.9328 | 2 | 6826.33 | 4314.84 | 3.23 | 7.94 | 2.000E-04 |
| T998 | SGADGKPGTMGKPTDDK | 581.2536 | 3 | 112.06 | 78.60 | 0.00 | 0.00 | 6.000E-04 |
| T1036 | DSKPSAGGPKPGDQKPTPGAGAPK | 582.0363 | 4 | 347.45 | 241.80 | 20.68 | 19.76 | 9.000E-04 |
| S1056 | PQAAGTISKPGESQKK | 569.6202 | 3 | 44798.65 | 23238.13 | 41.01 | 69.75 | 1.000E-04 |
| S1123 | SAAPSAPSDAKPDSK | 503.5591 | 3 | 167.03 | 103.75 | 0.00 | 0.00 | 2.000E-04 |
| S1254 | VGQSSAAAGGER | 585.2509 | 2 | 677.47 | 587.76 | 0.99 | 1.97 | 3.300E-03 |

**Table S1B. Tryptic phosphopeptides used for quantification of wild type light versus *norpA^P24^* light.**

| **phosphorylation site** | **peptide** | **m/z** | **charge** | **mean normalized abundance (wild type light)** | **SD (wild type light)** | **mean normalized abundance (*norpA^P24^* light)** | **SD (*norpA^P24^* light)** | **p-value** |
| --- | --- | --- | --- | --- | --- | --- | --- | --- |
| S721 | TKSFMR | 425.1882 | 2 | 88.31 | 49.84 | 82.49 | 91.22 | 8.69E-01 |
| T849 | KKTQKGDKDWNAIAR | 460.4873 | 4 | 24009.80 | 8153.99 | 12.78 | 15.53 | 1.00E-04 |
| T864 | KNTFASDPIGSK | 672.8133 | 2 | 40113.11 | 20182.35 | 719.85 | 355.10 | 1.00E-04 |
| S872 | KNTFASDPIGSKR | 500.9117 | 3 | 40036.01 | 28273.44 | 12.39 | 22.42 | 6.00E-04 |
| S936 | M(ox)AADEVSLADDEGAPNGEGEK | 1100.9334 | 2 | 4.25 | 12.75 | 4428.54 | 5998.44 | 4.18E-02 |
| S961 | SITSGGTGGGASM(ox)LAAAALR | 615.6239 | 3 | 1645.93 | 1204.03 | 26.73 | 43.70 | 1.00E-03 |
| S964 | SITSGGTGGGASM(ox)LAAAALR | 922.9328 | 2 | 6826.33 | 4314.84 | 11.08 | 21.41 | 2.00E-04 |
| T998 | SGADGKPGTMGKPTDDK | 581.2536 | 3 | 112.06 | 78.60 | 0.00 | 0.00 | 6.00E-04 |
| T1036 | DSKPSAGGPKPGDQKPTPGAGAPK | 582.0363 | 4 | 347.45 | 241.80 | 502.24 | 230.96 | 1.84E-01 |
| S1056 | PQAAGTISKPGESQKK | 569.6202 | 3 | 44798.65 | 23238.13 | 220.34 | 188.21 | 1.00E-04 |
| S1123 | SAAPSAPSDAKPDSK | 503.5591 | 3 | 167.03 | 103.75 | 0.00 | 0.00 | 2.00E-04 |
| S1254 | VGQSSAAAGGER | 585.2509 | 2 | 677.47 | 587.76 | 1.43 | 3.04 | 3.30E-03 |

**Table S1C. Tryptic phosphopeptides used for quantification of wild *trp^P365^*/+ dark versus wild type dark.**

| **phosphorylation site** | **peptide** | **m/z** | **charge** | **mean normalized abundance (*trp^P365^*/+ dark)** | **SD (*trp^P365^*/+ dark)** | **mean normalized abundance (wild type dark)** | **SD (wild type dark)** | **p-value** |
| --- | --- | --- | --- | --- | --- | --- | --- | --- |
| S721 | TKSFMR | 425.1882 | 2 | 47.06 | 26.98 | 41.45 | 34.57 | 7.06E-01 |
| T849 | KKTQKGDKDWNAIAR | 460.4873 | 4 | 378.09 | 240.56 | 92.68 | 46.31 | 3.00E-03 |
| T864 | KNTFASDPIGSK | 672.8133 | 2 | 1845.87 | 471.66 | 555.37 | 144.35 | 1.00E-04 |
| S872 | KNTFASDPIGSKR | 500.9117 | 3 | 1187.54 | 181.55 | 34.22 | 27.17 | 1.00E-04 |
| S936 | M(ox)AADEVSLADDEGAPNGEGEK | 1100.9334 | 2 | 1742.17 | 3194.83 | 11638.81 | 9498.77 | 9.20E-03 |
| T1036 | DSKPSAGGPKPGDQKPTPGAGAPK | 582.0363 | 4 | 36.95 | 27.50 | 20.68 | 19.76 | 1.69E-01 |
| S1056 | PQAAGTISKPGESQKK | 569.6202 | 3 | 2247.22 | 948.58 | 41.01 | 69.75 | 3.00E-04 |

**Table S1D. Chymotryptic phosphopeptides used for quantification of wild type light versus wild type dark.**

| **phosphorylation site** | **peptide** | **m/z** | **charge** | **mean normalized abundance (wild type light)** | **SD (light)** | **mean normalized abundance (wild type dark)** | **SD (dark)** | **p-value** |
| --- | --- | --- | --- | --- | --- | --- | --- | --- |
| S726 | MRKSMERAQTL | 477.5598 | 4 | 13569.44 | 3438.52 | 120.88 | 84.17 | 1,00E-04 |
| T849 | SSLAKVIGRKKTQKGDKDW | 557.0506 | 2 | 63024.07 | 22420.32 | 1937.52 | 674.60 | 1,00E-04 |
| T864 | NAIARKNTF | 557.7738 | 2 | 16875.23 | 7700.13 | 311.92 | 191.91 | 1,00E-04 |
| S872 | ASDPIGSKRSSM | 658.2887 | 4 | 9027.98 | 5910.91 | 5.54 | 12.91 | 1,00E-04 |
| S876 | ASDPIGSKRSSM | 658.2892 | 3 | 13495.94 | 9059.58 | 117.75 | 112.11 | 1,00E-04 |
| S936 | AADEVSLADDEGAPNGEGEKKPL | 798.0233 | 3 | 2280.45 | 718.06 | 214.77 | 122.05 | 2,00E-03 |
| S958 | DASGSKKSITSGGTGGGASM(ox)L | 655.6262 | 2 | 10687.46 | 3143.97 | 6.37 | 17.26 | 1,00E-04 |
| S961 | DASGSKKSITSGGTGGGASML | 650.2946 | 2 | 52983.04 | 15821.02 | 36.89 | 51.41 | 1,00E-04 |
| S982 | RASVKNVDEKSGADGKPGTM | 532.5028 | 4 | 13569.44 | 3438.52 | 120.88 | 84.17 | 1,00E-04 |

**Table S1E. Chymotryptic phosphopeptides used for quantification of wild type light versus *norpA^P24^* light.**

| **phosphorylation site** | **peptide** | **m/z** | **charge** | **mean normalized abundance (wild type light)** | **SD (light)** | **mean normalized abundance (*norpA^P24^* light)** | **SD (dark)** | **p-value** |
| --- | --- | --- | --- | --- | --- | --- | --- | --- |
| S726 | MRKSMERAQTL | 477.5598 | 3 | 2108.26 | 1077.67 | 19.07 | 39.57 | 1.00E-04 |
| T849 | SSLAKVIGRKKTQKGDKDW | 557.0506 | 4 | 13569.44 | 3438.52 | 119.21 | 100.38 | 1.00E-04 |
| T864 | NAIARKNTF | 557.7738 | 2 | 63024.07 | 22420.32 | 2009.78 | 876.05 | 1.00E-04 |
| S872 | ASDPIGSKRSSM | 658.2887 | 2 | 16875.23 | 7700.13 | 379.44 | 222.12 | 1.00E-04 |
| S876 | ASDPIGSKRSSM | 658.2892 | 2 | 30706.18 | 5917.47 | 2554.44 | 1082.51 | 1.00E-04 |
| S936 | M(ox)RKKM(ox)AADEVSLADDEGAPNGEGEKKPL | 775.3601 | 4 | 1363.94 | 387.09 | 20792.82 | 7577.58 | 1.00E-04 |
| S958 | DASGSKKSITSGGTGGGASM(ox)L | 655.6262 | 3 | 2280.45 | 718.06 | 167.82 | 115.09 | 1.00E-04 |
| S961 | DASGSKKSITSGGTGGGASML | 650.2946 | 3 | 4513.93 | 1139.27 | 167.30 | 81.48 | 1.00E-04 |
| S982 | RASVKNVDEKSGADGKPGTM | 532.5028 | 4 | 359374.12 | 145467.11 | 7367.12 | 4210.03 | 1.00E-04 |

**Table S1F. Chymotryptic phosphopeptides used for quantification of wild *trp^P365^*/+ dark versus wild type dark.**

| **phosphorylation site** | **peptide** | **m/z** | **charge** | **mean normalized abundance (*trp^P365^*/+ dark)** | **SD (light)** | **mean normalized abundance (wild type dark)** | **SD (dark)** | **p-value** |
| --- | --- | --- | --- | --- | --- | --- | --- | --- |
| T849 | SSLAKVIGRKKTQKGDKDW | 557.0506 | 4 | 4588.34 | 4125.08 | 120.88 | 84.17 | 5.00E-03 |
| T864 | NAIARKNTF | 557.7738 | 2 | 6180.30 | 1469.85 | 1937.52 | 674.60 | 1.00E-04 |
| S872 | ASDPIGSKRSSM | 658.2887 | 2 | 1138.26 | 1097.73 | 311.92 | 191.91 | 4.08E-02 |
| S876 | ASDPIGSKRSSM | 658.2892 | 2 | 3198.05 | 1209.66 | 1035.23 | 528.52 | 2.00E-04 |
| S936 | AADEVSLADDEGAPNGEGEKKPL | 798.0233 | 3 | 22497.01 | 11410.69 | 35985.71 | 29188.32 | 2.15E-01 |
| S958 | DASGSKKSITSGGTGGGASM(ox)L | 655.6262 | 3 | 349.47 | 147.61 | 214.77 | 122.05 | 5.10E-02 |
| S961 | DASGSKKSITSGGTGGGASML | 650.2946 | 3 | 863.08 | 457.36 | 120.98 | 111.70 | 2.00E-04 |
| S982 | RASVKNVDEKSGADGKPGTM | 532.5028 | 4 | 62953.91 | 30034.16 | 7446.77 | 2368.54 | 1.00E-04 |
