# Supplementary material for: Phosphorylation of the Drosophila Transient Receptor Potential Ion Channel Is Regulated by the Phototransduction Cascade and Involves Several Protein Kinases and Phosphatases
Source: PLoS One. 2013 Sep 9;8(9):e73787. doi: 10.1371/journal.pone.0073787 (PMC3767779; doi:10.1371/journal.pone.0073787)
Supplement: Table S2 — Listing of flies used for the candidate screen to identify kinases and phosphatases of TRP and detailed results. (DOCX) [file pone.0073787.s005.docx]

| **Kinase/**  **Phosphatase** | **Genotype** | **Phosphory-lation site** | **Phosphorylation in comparison to wild type flies [%]** | | | | |
| --- | --- | --- | --- | --- | --- | --- | --- |
|  |  |  | **1^st^ round** | **2^nd^ round** | **3^rd^ round** | **Mean** | **P-value** |
| w Oregon R | w; +; + | Thr^849^ (light) | - | - | - | 100 | - |
|  |  | Thr^849^ (dark) | - | - | - | 18.72 | <0.0001 |
|  |  | Thr^864^ (light) | - | - | - | 100 | - |
|  |  | Thr^864^ (dark) | - | - | - | 33.95 | <0.0001 |
| Activated Cdc42 kinase | y[1] w[67c23]; +; P{w[+mC] y[+mDint2]=EPgy2}Ack[EY09374] | Thr^849^ (light) | 142 | - | - | - | - |
|  |  | Thr^849^ (dark) | 6 | - | - | - | - |
|  |  | Thr^864^ (light) | 92 | - | - | - | - |
|  |  | Thr^864^ (dark) | 14 | - | - | - | - |
| Akt1 | y[1] w[67c23]; P{w[+mC] y[+mDint2]=EPgy2}Akt1[EY10012]/TM3, Sb[1] Ser[1] | Thr^849^ (light) | 124 | - | - | - | - |
|  |  | Thr^849^ (dark) | 1 | - | - | - | - |
|  |  | Thr^864^ (light) | 83 | - | - | - | - |
|  |  | Thr^864^ (dark) | 1 | - | - | - | - |
| alphabet | y[1] w[67c23]; +; Mi{ET1}alph[MB01683] | Thr^849^ (light) | 136 | - | - | - | - |
|  |  | Thr^849^ (dark) | 4 | - | - | - | - |
|  |  | Thr^864^ (light) | 108 | - | - | - | - |
|  |  | Thr^864^ (dark) | 14 | - | - | - | - |
| Arginine kinase | y[1]; P{y[+mDint2] w[BR.E.BR]=SUPor-P}Argk[KG10020] ry[506] | Thr^849^ (light) | 116 | - | - | - | - |
|  |  | Thr^849^ (dark) | 2 | - | - | - | - |
|  |  | Thr^864^ (light) | 87 | - | - | - | - |
|  |  | Thr^864^ (dark) | 6 | - | - | - | - |
| Arginine kinase | w[*]; +; PBac{GAL4D,EYFP} Argk[PL00418] P{w[+mW.hs]= FRT(w[hs])}2A P{ry[+t7.2]= neoFRT}82B/rh1>gal4 | Thr^849^ (light) | 206 | 166 | 106 | 159.27 | 0.1794 |
|  |  | Thr^849^ (dark) | 7 | 1 | 0 | 2.91 | 0.1173 |
|  |  | Thr^864^ (light) | 94 | - | - | - | - |
|  |  | Thr^864^ (dark) | 27 | - | - | - | - |
| Atypical protein kinase C | y[1] v[1]; +; P{y[+t7.7] v[+t1.8]=TRiP.JF01966}attP2/rh1>gal4 | Thr^849^ (light) | 101 | - | - | - | - |
|  |  | Thr^849^ (dark) | 1 | - | - | - | - |
|  |  | Thr^864^ (light) | 85 | - | - | - | - |
|  |  | Thr^864^ (dark) | 7 | - | - | - | - |
| cAMP-dependent protein kinase 1 | +; Pka-C1[B10]; + | Thr^849^ (light) | 88 | - | - | - | - |
|  |  | Thr^849^ (dark) | 1 | - | - | - | - |
|  |  | Thr^864^ (light) | 117 | - | - | - | - |
|  |  | Thr^864^ (dark) | 8 | - | - | - | - |

| **Kinase/**  **Phosphatase** | **Genotype** | **Phosphory-lation site** | **Phosphorylation in comparison to wild type flies [%]** | | | | |
| --- | --- | --- | --- | --- | --- | --- | --- |
|  |  |  | **1^st^ round** | **2^nd^ round** | **3^rd^ round** | **Mean** | **P-value** |
| cAMP-dependent protein kinase 3 | y[1] w[67c23]; +; P{w[+mC] y[+mDint2]=EPgy2}Pka-C3[EY02687] | Thr^849^ (light) | 88 | - | - | - | - |
|  |  | Thr^849^ (dark) | 1 | - | - | - | - |
|  |  | Thr^864^ (light) | 149 | - | - | - | - |
|  |  | Thr^864^ (dark) | 13 | - | - | - | - |
| Caki | y[1] w[67c23]; +; P{w[+mC] y[+mDint2]=EPgy2}CASK[EY07081] | Thr^849^ (light) | 153 | - | - | - | - |
|  |  | Thr^849^ (dark) | 12 | - | - | - | - |
|  |  | Thr^864^ (light) | 59 | - | - | - | - |
|  |  | Thr^864^ (dark) | 2 | - | - | - | - |
| Calcium/-calmodulin-dependent protein kinase I | y[1] w[67c23]; +; +; P{w[+mC] y[+mDint2]=EPgy2}CaMKI[EY07197] | Thr^849^ (light) | 102 | - | - | - | - |
|  |  | Thr^849^ (dark) | 2 | - | - | - | - |
|  |  | Thr^864^ (light) | 92 | - | - | - | - |
|  |  | Thr^864^ (dark) | 8 | - | - | - | - |
| Casein kinase Iα | w[1118] P{w[+mC]=EP}CkIalpha[EP1555] EP1555[EP1555]; +; + | Thr^849^ (light) | 70 | - | - | - | - |
|  |  | Thr^849^ (dark) | 1 | - | - | - | - |
|  |  | Thr^864^ (light) | 17 | 39 | 32 | 29.31 | 0.0078 |
|  |  | Thr^864^ (dark) | 4 | 6 | 3 | 4.47 | 0.0427 |
| Casein kinase IIα | y[1] w[*]; +; P{w[+mC]=UAS-CkIIalpha.Tik}T1/rh1>gal4 | Thr^849^ (light) | 108 | - | - | - | - |
|  |  | Thr^849^ (dark) | 1 | - | - | - | - |
|  |  | Thr^864^ (light) | 163 | - | - | - | - |
|  |  | Thr^864^ (dark) | 9 | - | - | - | - |
| CG10376 | y[1] w[*]; P{w[+mC]=EP}CG10376[G2887]; + | Thr^849^ (light) | 117 | - | - | - | - |
|  |  | Thr^849^ (dark) | 3 | - | - | - | - |
|  |  | Thr^864^ (light) | 74 | - | - | - | - |
|  |  | Thr^864^ (dark) | 1 | - | - | - | - |
| CG17746 | y[1] w[*]; +; P{w[+mC]=EP}CG17746[G4827] | Thr^849^ (light) | 90 | - | - | - | - |
|  |  | Thr^849^ (dark) | 2 | - | - | - | - |
|  |  | Thr^864^ (light) | 118 | - | - | - | - |
|  |  | Thr^864^ (dark) | 14 | - | - | - | - |
| CG7115 | [1] w[67c23]; P{y[+mDint2] w[BR.E.BR]=SUPor-P}CG7115[KG02655]; + | Thr^849^ (light) | 113 | - | - | - | - |
|  |  | Thr^849^ (dark) | 3 | - | - | - | - |
|  |  | Thr^864^ (light) | 132 | - | - | - | - |
|  |  | Thr^864^ (dark) | 15 | - | - | - | - |

| **Kinase/**  **Phosphatase** | **Genotype** | **Phosphory-lation site** | **Phosphorylation in comparison to wild type flies [%]** | | | | |
| --- | --- | --- | --- | --- | --- | --- | --- |
|  |  |  | **1^st^ round** | **2^nd^ round** | **3^rd^ round** | **Mean** | **P-value** |
| CG7766 | w[*] P{w[+mC]=EP}CG7766[G104]; +; + | Thr^849^ (light) | 49 | 160 | 105 | 104.16 | 0.9085 |
|  |  | Thr^849^ (dark) | 1 | 1 | 1 | 0.83 | 0.0595 |
|  |  | Thr^864^ (light) | 56 | - | - | - | - |
|  |  | Thr^864^ (dark) | 8 | - | - | - | - |
| Corkscrew | csw[lf] P{w[+mW.hs]=FRT(w[hs])}101/FM7c; +; + | Thr^849^ (light) | 106 | - | - | - | - |
|  |  | Thr^849^ (dark) | 1 | - | - | - | - |
|  |  | Thr^864^ (light) | 85 | - | - | - | - |
|  |  | Thr^864^ (dark) | 7 | - | - | - | - |
| Downstream of raf1 (MEK) | w[*] P{w[+mC]=EP}Dsor1[G9740]; +;+ | Thr^849^ (light) | 61 | - | - | - | - |
|  |  | Thr^849^ (dark) | 1 | - | - | - | - |
|  |  | Thr^864^ (light) | 74 | - | - | - | - |
|  |  | Thr^864^ (dark) | 5 | - | - | - | - |
| Eye-protein kinase C | w[*]; inaC[P209]; + | Thr^849^ (light) | 17 | 15 | 12 | 14.59 | 0.0003 |
|  |  | Thr^849^ (dark) | 6 | 7 | 6 | 5.89 | 0.0655 |
|  |  | Thr^864^ (light) | 101 | - | - | - | - |
|  |  | Thr^864^ (dark) | 41 | - | - | - | - |
| Flapwing | y[2] cho[2] flw[1]; +; + | Thr^849^ (light) | 156 | - | - | - | - |
|  |  | Thr^849^ (dark) | 6 | - | - | - | - |
|  |  | Thr^864^ (light) | 105 | - | - | - | - |
|  |  | Thr^864^ (dark) | 21 | - | - | - | - |
| Foraging | y[1] w[67c23]; P{w[+mC] y[+mDint2]=EPgy2}for[EY10922]; + | Thr^849^ (light) | 54 | - | - | - | - |
|  |  | Thr^849^ (dark) | 1 | - | - | - | - |
|  |  | Thr^864^ (light) | 58 | - | - | - | - |
|  |  | Thr^864^ (dark) | 5 | - | - | - | - |
| Fps oncogene analog | w[1118]; +; Mi{ET1}Fps85D[MB08538] | Thr^849^ (light) | 29 | 184 | 170 | 127.88 | 0.6301 |
|  |  | Thr^849^ (dark) | 1 | 1 | 2 | 1.70 | 0.0989 |
|  |  | Thr^864^ (light) | 59 | - | - | - | - |
|  |  | Thr^864^ (dark) | 1 | - | - | - | - |
| Germinal centre kinase III | y[1] w[67c23]; +; P{w[+mC] y[+mDint2]=EPgy2}GckIII[EY05076]/TM3, Sb[1] Ser[1] | Thr^849^ (light) | 78 | - | - | - | - |
|  |  | Thr^849^ (dark) | 1 | - | - | - | - |
|  |  | Thr^864^ (light) | 56 | - | - | - | - |
|  |  | Thr^864^ (dark) | 4 | - | - | - | - |

| **Kinase/**  **Phosphatase** | **Genotype** | **Phosphory-lation site** | **Phosphorylation in comparison to wild type flies [%]** | | | | |
| --- | --- | --- | --- | --- | --- | --- | --- |
|  |  |  | **1^st^ round** | **2^nd^ round** | **3^rd^ round** | **Mean** | **P-value** |
| Genghis khan | y[1] w[67c23]; P{w[+mC] y[+mDint2]=EPgy2}gek[EY02416]; + | Thr^849^ (light) | 105 | - | - | - | - |
|  |  | Thr^849^ (dark) | 1 | - | - | - | - |
|  |  | Thr^864^ (light) | 89 | - | - | - | - |
|  |  | Thr^864^ (dark) | 9 | - | - | - | - |
| Gilgamesh | y[1] w[1118]; +; PBac{w[+mC]=5HPw[+]}gish[B184] | Thr^849^ (light) | 231 | 174 | 115 | 173.88 | 0.1576 |
|  |  | Thr^849^ (dark) | 2 | 1 | 1 | 1.13 | 0.0929 |
|  |  | Thr^864^ (light) | 87 | - | - | - | - |
|  |  | Thr^864^ (dark) | 9 | - | - | - | - |
| Gilgamesh | y[1] w[67c23]; +; P{w[+mC] y[+mDint2]=EPgy2}gish[EY06451] | Thr^849^ (light) | 179 | - | - | - | - |
|  |  | Thr^849^ (dark) | 2 | - | - | - | - |
|  |  | Thr^864^ (light) | 110 | - | - | - | - |
|  |  | Thr^864^ (dark) | 13 | - | - | - | - |
| G protein-coupled receptor kinase 2 | w[1118]; +; Mi{ET1}Gprk2[MB08070] | Thr^849^ (light) | 109 | - | - | - | - |
|  |  | Thr^849^ (dark) | 3 | - | - | - | - |
|  |  | Thr^864^ (light) | 116 | - | - | - | - |
|  |  | Thr^864^ (dark) | 21 | - | - | - | - |
| Grapes | w[1118]; Mi{ET1}grp[MB08323]; + | Thr^849^ (light) | 117 | - | - | - | - |
|  |  | Thr^849^ (dark) | 3 | - | - | - | - |
|  |  | Thr^864^ (light) | 95 | - | - | - | - |
|  |  | Thr^864^ (dark) | 9 | - | - | - | - |
| Hopscotch | y[1] P{y[+mDint2] w[BR.E.BR]=SUPor-P}hop[KG01990]; +; + | Thr^849^ (light) | 132 | - | - | - | - |
|  |  | Thr^849^ (dark) | 3 | - | - | - | - |
|  |  | Thr^864^ (light) | 119 | - | - | - | - |
|  |  | Thr^864^ (dark) | 15 | - | - | - | - |
| Leukocyte-antigen-related-like | w[1118]; Mi{ET1}Lar[MB03324]; + | Thr^849^ (light) | 97 | - | - | - | - |
|  |  | Thr^849^ (dark) | 4 | - | - | - | - |
|  |  | Thr^864^ (light) | 101 | - | - | - | - |
|  |  | Thr^864^ (dark) | 14 | - | - | - | - |
| lethal (1) G0232 | w[1118] PBac{w[+mC]=WH}l(1)G0232[f06600]; ?/CyO; + | Thr^849^ (light) | 117 | - | - | - | - |
|  |  | Thr^849^ (dark) | 3 | - | - | - | - |
|  |  | Thr^864^ (light) | 112 | - | - | - | - |
|  |  | Thr^864^ (dark) | 15 | - | - | - | - |

| **Kinase/**  **Phosphatase** | **Genotype** | **Phosphory-lation site** | **Phosphorylation in comparison to wild type flies [%]** | | | | |
| --- | --- | --- | --- | --- | --- | --- | --- |
|  |  |  | **1^st^ round** | **2^nd^ round** | **3^rd^ round** | **Mean** | **P-value** |
| Licorne | w[67c23] P{w[+mC]=lacW}lic[G0252]/FM7c; +; + | Thr^849^ (light) | 88 | - | - | - | - |
|  |  | Thr^849^ (dark) | 8 | - | - | - | - |
|  |  | Thr^864^ (light) | 23 | 23 | 20 | 21.96 | 0.0002 |
|  |  | Thr^864^ (dark) | 3 | 1 | 1 | 1.46 | 0.0009 |
| Lightoid | w[1118]; Mi{ET1}ltd[MB03690]; + | Thr^849^ (light) | 91 | - | - | - | - |
|  |  | Thr^849^ (dark) | 3 | - | - | - | - |
|  |  | Thr^864^ (light) | 104 | - | - | - | - |
|  |  | Thr^864^ (dark) | 16 | - | - | - | - |
| MAP kinase activated protein-kinase-2 | w[*] P{w[+mC]=EP}MAPk-Ak2[G265]; +; + | Thr^849^ (light) | 124 | - | - | - | - |
|  |  | Thr^849^ (dark) | 1 | - | - | - | - |
|  |  | Thr^864^ (light) | 132 | - | - | - | - |
|  |  | Thr^864^ (dark) | 2 | - | - | - | - |
| MAP kinase kinase 4 | w[1118]; +; PBac{w[+mC]=RB}Mkk4[e01485]/TM6B, Tb[1] | Thr^849^ (light) | 104 | - | - | - | - |
|  |  | Thr^849^ (dark) | 2 | - | - | - | - |
|  |  | Thr^864^ (light) | 120 | - | - | - | - |
|  |  | Thr^864^ (dark) | 4 | - | - | - | - |
| Mekk1 | y[1] w[67c23]; +; P{w[+mC] y[+mDint2]=EPgy2}Mekk1[EY11461] | Thr^849^ (light) | 151 | - | - | - | - |
|  |  | Thr^849^ (dark) | 2 | - | - | - | - |
|  |  | Thr^864^ (light) | 114 | - | - | - | - |
|  |  | Thr^864^ (dark) | 11 | - | - | - | - |
| Mpk2 | w[*]; +; P{ry[+t7.2]=neoFRT}82B Mpk2[1] | Thr^849^ (light) | 76 | - | - | - | - |
|  |  | Thr^849^ (dark) | 2 | - | - | - | - |
|  |  | Thr^864^ (light) | 97 | - | - | - | - |
|  |  | Thr^864^ (dark) | 3 | - | - | - | - |
| MPPE | y[1] w[67c23]; P{y[+mDint2] w[BR.E.BR]=SUPor-P}Mppe[KG05213]; + | Thr^849^ (light) | 118 | - | - | - | - |
|  |  | Thr^849^ (dark) | 1 | - | - | - | - |
|  |  | Thr^864^ (light) | 42 | 46 | 25 | 37.59 | 0.0110 |
|  |  | Thr^864^ (dark) | 2 | 3 | 4 | 2.84 | 0.0377 |
| Microtubule star | +; +; mts[XE-2258]/CyO, P{ry[+t7.2]=sevRas1.V12}FK1 | Thr^849^ (light) | 98 | - | - | - | - |
|  |  | Thr^849^ (dark) | 6 | - | - | - | - |
|  |  | Thr^864^ (light) | 147 | - | - | - | - |
|  |  | Thr^864^ (dark) | 14 | - | - | - | - |

| **Kinase/**  **Phosphatase** | **Genotype** | **Phosphory-lation site** | **Phosphorylation in comparison to wild type flies [%]** | | | | |
| --- | --- | --- | --- | --- | --- | --- | --- |
|  |  |  | **1^st^ round** | **2^nd^ round** | **3^rd^ round** | **Mean** | **P-value** |
| Microtubule star | y[1] w[67c23]; P{w[+mC] y[+mDint2]=EPgy2}mts[EY12638]; + | Thr^849^ (light) | 100 | - | - | - | - |
|  |  | Thr^849^ (dark) | 2 | - | - | - | - |
|  |  | Thr^864^ (light) | 51 |  |  |  |  |
|  |  | Thr^864^ (dark) | 7 |  |  |  |  |
| Mitogen-activated protein kinase phos-phatase 3 | y[1] w[67c23]; +; P{w[+mC] y[+mDint2]=EPgy2}Mkp3[EY02009a] | Thr^849^ (light) | 107 | - | - | - | - |
|  |  | Thr^849^ (dark) | 2 | - | - | - | - |
|  |  | Thr^864^ (light) | 75 | - | - | - | - |
|  |  | Thr^864^ (dark) | 10 | - | - | - | - |
| Mushroom bodies tiny | y[1] w[67c23] P{w[+mC] y[+mDint2]=EPgy2}mbt[EY08341]; +; + | Thr^849^ (light) | 164 | - | - | - | - |
|  |  | Thr^849^ (dark) | 3 | - | - | - | - |
|  |  | Thr^864^ (light) | 166 | - | - | - | - |
|  |  | Thr^864^ (dark) | 11 | - | - | - | - |
| Neither inactivation nor after-potential C | w[*]; ninaC[3]; + | Thr^849^ (light) | 71 | - | - | - | - |
|  |  | Thr^849^ (dark) | 3 | - | - | - | - |
|  |  | Thr^864^ (light) | 69 | - | - | - | - |
|  |  | Thr^864^ (dark) | 2 | - | - | - | - |
| P38b | y[1] w[67c23]; P{w[+mC] y[+mDint2]=EPgy2}p38b[EY11174]; + | Thr^849^ (light) | 136 | - | - | - | - |
|  |  | Thr^849^ (dark) | 25 | - | - | - | - |
|  |  | Thr^864^ (light) | 72 | - | - | - | - |
|  |  | Thr^864^ (dark) | 30 | - | - | - | - |
| Pak3 | y[1]; +; ry[506] P{y[+mDint2] w[BR.E.BR]=SUPor-P}Pak3[KG00430] | Thr^849^ (light) | 141 | - | - | - | - |
|  |  | Thr^849^ (dark) | 4 | - | - | - | - |
|  |  | Thr^864^ (light) | 89 | - | - | - | - |
|  |  | Thr^864^ (dark) | 11 | - | - | - | - |
| Par-1 | y[1] w[67c23]; P{y[+t7.7] w[+mC]=wHy}mei-W68[DG23501] par-1[DG23501]; + | Thr^849^ (light) | 133 | - | - | - | - |
|  |  | Thr^849^ (dark) | 3 | - | - | - | - |
|  |  | Thr^864^ (light) | 65 | - | - | - | - |
|  |  | Thr^864^ (dark) | 9 | - | - | - | - |
| PTEN-induced putative kinase 1 | w[*] P{w[+mC]=EP}Pink1[G900]; +; + | Thr^849^ (light) | 118 | - | - | - | - |
|  |  | Thr^849^ (dark) | 3 | - | - | - | - |
|  |  | Thr^864^ (light) | 76 | - | - | - | - |
|  |  | Thr^864^ (dark) | 9 | - | - | - | - |

| **Kinase/**  **Phosphatase** | **Genotype** | **Phosphory-lation site** | **Phosphorylation in comparison to wild type flies [%]** | | | | |
| --- | --- | --- | --- | --- | --- | --- | --- |
|  |  |  | **1^st^ round** | **2^nd^ round** | **3^rd^ round** | **Mean** | **P-value** |
| Protein kinase-like 17E | y[1] w[67c23] P{w[+mC] y[+mDint2]=EPgy2}Pk17E[EY10051]; +; + | Thr^849^ (light) | 114 | - | - | - | - |
|  |  | Thr^849^ (dark) | 2 | - | - | - | - |
|  |  | Thr^864^ (light) | 95 | - | - | - | - |
|  |  | Thr^864^ (dark) | 10 | - | - | - | - |
| Phospho-inositide-dependent kinase 1 | w[1118]; +; P{w[+mGT]=GT1}Pdk1[BG02759] | Thr^849^ (light) | 145 | - | - | - | - |
|  |  | Thr^849^ (dark) | 1 | - | - | - | - |
|  |  | Thr^864^ (light) | 207 | 132 | 90 | 142.68 | 0.3391 |
|  |  | Thr^864^ (dark) | 8 | 9 | 5 | 7.59 | 0.0501 |
| Protein kinase at 92B | w[1118]; +; Mi{ET1}Pk92B[MB06487] | Thr^849^ (light) | 48 | 48 | 80 | 58.68 | 0.0602 |
|  |  | Thr^849^ (dark) | 1 | 1 | 2 | 1.16 | 0.0655 |
|  |  | Thr^864^ (light) | 96 | - | - | - | - |
|  |  | Thr^864^ (dark) | 15 | - | - | - | - |
| Protein C kinase 53 E | w[1118]; Mi{ET1}Pkc53E[MB02781]; + | Thr^849^ (light) | 43 | 52 | 22 | 38.88 | 0.0202 |
|  |  | Thr^849^ (dark) | 1 | 0 | 1 | 0.74 | 0.0091 |
|  |  | Thr^864^ (light) | 59 | - | - | - | - |
|  |  | Thr^864^ (dark) | 2 | - | - | - | - |
| PKC53E (RNAi) | y[1] v[1]; +; P{y[+t7.7] v[+t1.8]=TRiP.JF02641}attP2/rh1>gal4 | Thr^849^ (light) | 60 | - | - | - | - |
|  |  | Thr^849^ (dark) | 1 | - | - | - | - |
|  |  | Thr^864^ (light) | 91 | - | - | - | - |
|  |  | Thr^864^ (dark) | 6 | - | - | - | - |
| primo-1 | y[1] w[67c23]; +; P{w[+mC] y[+mDint2]=EPgy2}primo-2[EY21742] primo-1[EY21742] | Thr^849^ (light) | 121 | - | - | - | - |
|  |  | Thr^849^ (dark) | 1 | - | - | - | - |
|  |  | Thr^864^ (light) | 108 | - | - | - | - |
|  |  | Thr^864^ (dark) | 7 | - | - | - | - |
| primo-2 | w[1118]; +; PBac{w[+mC]=WH}primo-1[f03836] primo-2[f03836] | Thr^849^ (light) | 124 | - | - | - | - |
|  |  | Thr^849^ (dark) | 3 | - | - | - | - |
|  |  | Thr^864^ (light) | 172 | - | - | - | - |
|  |  | Thr^864^ (dark) | 12 | - | - | - | - |
| PRL-1 | y[1] w[67c23]; P{w[+mC] y[+mDint2]=EPgy2}PRL-1[EY11934]; + | Thr^849^ (light) | 150 | - | - | - | - |
|  |  | Thr^849^ (dark) | 7 | - | - | - | - |
|  |  | Thr^864^ (light) | 41 | 109 | 66 | 72.12 | 0.2319 |
|  |  | Thr^864^ (dark) | 4 | 13 | 4 | 6.78 | 0.0151 |

| **Kinase/**  **Phosphatase** | **Genotype** | **Phosphory-lation site** | **Phosphorylation in comparison to wild type flies [%]** | | | | |
| --- | --- | --- | --- | --- | --- | --- | --- |
|  |  |  | **1^st^ round** | **2^nd^ round** | **3^rd^ round** | **Mean** | **P-value** |
| Protein C kinase 98E | w[1118]; +; PBac{w[+mC]=WH}Pkc98E[f06221]/TM6B, Tb[1] | Thr^849^ (light) | 96 | - | - | - | - |
|  |  | Thr^849^ (dark) | 2 | - | - | - | - |
|  |  | Thr^864^ (light) | 96 | - | - | - | - |
|  |  | Thr^864^ (dark) | 13 | - | - | - | - |
| Protein C kinase 98E | y[1] v[1]; +; P{y[+t7.7] v[+t1.8]=TRiP.JF02470}attP2/rh1>gal4 | Thr^849^ (light) | 108 | - | - | - | - |
|  |  | Thr^849^ (dark) | 1 | - | - | - | - |
|  |  | Thr^864^ (light) | 110 | - | - | - | - |
|  |  | Thr^864^ (dark) | 7 | - | - | - | - |
| Protein kinase related to protein kinase N | y[1] v[1]; +; P{y[+t7.7] v[+t1.8]=TRiP.JF02970}attP2/rh1>gal4 | Thr^849^ (light) | 99 | - | - | - | - |
|  |  | Thr^849^ (dark) | 1 | - | - | - | - |
|  |  | Thr^864^ (light) | 78 | - | - | - | - |
|  |  | Thr^864^ (dark) | 5 | - | - | - | - |
| Protein Kinase D | y[1] w[67c23]; +; Mi{ET1}PKD[MB00674] | Thr^849^ (light) | 88 | - | - | - | - |
|  |  | Thr^849^ (dark) | 4 | - | - | - | - |
|  |  | Thr^864^ (light) | 139 | - | - | - | - |
|  |  | Thr^864^ (dark) | 27 | - | - | - | - |
| Protein phosphatase 1 at 87B | w[1118]; +; P{w[+mGT]=GT1}Pp1-87B[BG00793] | Thr^849^ (light) | 99 | - | - | - | - |
|  |  | Thr^849^ (dark) | 2 | - | - | - | - |
|  |  | Thr^864^ (light) | 89 | - | - | - | - |
|  |  | Thr^864^ (dark) | 5 | - | - | - | - |
| Protein phosphatase 1α at 96A | +; +; ry[506] e[1] Pp1alpha-96A[2]/TM6B, P{w[+mW.hs]=Ubi-GFP.S65T}PAD2, Tb[1] | Thr^849^ (light) | 90 | - | - | - | - |
|  |  | Thr^849^ (dark) | 1 | - | - | - | - |
|  |  | Thr^864^ (light) | 87 | - | - | - | - |
|  |  | Thr^864^ (dark) | 7 | - | - | - | - |
| Protein phosphatase 2A at 29B | w[1118]; P{w[+mC]=EP}Pp2A-29B[EP2332]/CyO; + | Thr^849^ (light) | 75 | - | - | - | - |
|  |  | Thr^849^ (dark) | 1 | - | - | - | - |
|  |  | Thr^864^ (light) | 68 | - | - | - | - |
|  |  | Thr^864^ (dark) | 9 | - | - | - | - |
| Protein phosphatase 2B at 14D | w[67c23] P{y[+m8]=Mae-UAS.6.11}Pp2B-14D[GG01028]; +; rh1-Gal4 | Thr^849^ (light) | 132 | - | - | - | - |
|  |  | Thr^849^ (dark) | 8 | - | - | - | - |
|  |  | Thr^864^ (light) | 122 | - | - | - | - |
|  |  | Thr^864^ (dark) | 16 | - | - | - | - |

| **Kinase/**  **Phosphatase** | **Genotype** | **Phosphory-lation site** | **Phosphorylation in comparison to wild type flies [%]** | | | | |
| --- | --- | --- | --- | --- | --- | --- | --- |
|  |  |  | **1^st^ round** | **2^nd^ round** | **3^rd^ round** | **Mean** | **P-value** |
| Protein phosphatase 19C | w[*] P{w[+mC]=EP}Pp4-19C[G11307]/FM6, w[*]; +; + | Thr^849^ (light) | 100 | - | - | - | - |
|  |  | Thr^849^ (dark) | 4 | - | - | - | - |
|  |  | Thr^864^ (light) | 89 | - | - | - | - |
|  |  | Thr^864^ (dark) | 13 | - | - | - | - |
| Protein phosphatase D3 | y[1] w[67c23]; +; P{w[+mC] y[+mDint2]=EPgy2}PpD3[EY02912] | Thr^849^ (light) | 168 | - | - | - | - |
|  |  | Thr^849^ (dark) | 3 | - | - | - | - |
|  |  | Thr^864^ (light) | 80 | - | - | - | - |
|  |  | Thr^864^ (dark) | 9 | - | - | - | - |
| Protein tyrosine phosph-atase-ERK/ Enhancer of Ras1 | w[1118]; PBac{w[+mC]=WH}PTP-ER[f02707]; + | Thr^849^ (light) | 154 | - | - | - | - |
|  |  | Thr^849^ (dark) | 4 | - | - | - | - |
|  |  | Thr^864^ (light) | 81 | - | - | - | - |
|  |  | Thr^864^ (dark) | 13 | - | - | - | - |
| Pyruvate dehydro-genase kinase | y[1] w[67c23]; P{w[+mC] y[+mDint2]=EPgy2}Pdk[EY01879]; + | Thr^849^ (light) | 144 | - | - | - | - |
|  |  | Thr^849^ (dark) | 0 | - | - | - | - |
|  |  | Thr^864^ (light) | 130 | - | - | - | - |
|  |  | Thr^864^ (dark) | 3 | - | - | - | - |
| Pyruvate dehydro-genase phosphatase | w[1118] PBac{w[+mC]=RB}Pdp[e02351]; +; + | Thr^849^ (light) | 108 | - | - | - | - |
|  |  | Thr^849^ (dark) | 2 | - | - | - | - |
|  |  | Thr^864^ (light) | 127 | - | - | - | - |
|  |  | Thr^864^ (dark) | 21 | - | - | - | - |
| Retinal degener-ation C | w; rdgC[306]; + | Thr^849^ (light) | 101 | - | - | - | - |
|  |  | Thr^849^ (dark) | 9 | - | - | - | - |
|  |  | Thr^864^ (light) | 130 | - | - | - | - |
|  |  | Thr^864^ (dark) | 42 | - | - | - | - |
| Rolled | +; rl[1]; + | Thr^849^ (light) | 260 | 304 | 264 | 275.92 | 0.0064 |
|  |  | Thr^849^ (dark) | 0 | 1 | 1 | 0.72 | 0.0240 |
|  |  | Thr^864^ (light) | 120 | - | - | - | - |
|  |  | Thr^864^ (dark) | 1 | - | - | - | - |

| **Kinase/**  **Phosphatase** | **Genotype** | **Phosphory-lation site** | **Phosphorylation in comparison to wild type flies [%]** | | | | |
| --- | --- | --- | --- | --- | --- | --- | --- |
|  |  |  | **1^st^ round** | **2^nd^ round** | **3^rd^ round** | **Mean** | **P-value** |
| RPS6-p70-protein kinase | y[1] w[*]; +; S6k[l-1]/TM6B, P{y[+t7.7] ry[+t7.2]=Car20y}TPN1, Tb[1] | Thr^849^ (light) | 100 | - | - | - | - |
|  |  | Thr^849^ (dark) | 11 | - | - | - | - |
|  |  | Thr^864^ (light) | 93 | - | - | - | - |
|  |  | Thr^864^ (dark) | 10 | - | - | - | - |
| Saxophone | y[1] w[*]; P{w[+mW.hs]=FRT(w[hs])}G13 sax[4]/SM6a; + | Thr^849^ (light) | 297 | 635 | 373 | 434.82 | 0.0820 |
|  |  | Thr^849^ (dark) | 1 | 3 | 2 | 2.05 | 0.0750 |
|  |  | Thr^864^ (light) | 101 | - | - | - | - |
|  |  | Thr^864^ (dark) | 3 | - | - | - | - |
| Shaggy | w[1118]; P{w[+mC]=UAS-sgg.A81T}MB2/rh1>gal4 | Thr^849^ (light) | 110 | - | - | - | - |
|  |  | Thr^849^ (dark) | 2 | - | - | - | - |
|  |  | Thr^864^ (light) | 125 | - | - | - | - |
|  |  | Thr^864^ (dark) | 14 | - | - | - | - |
| Shaggy | w[1118]; P{w[+mC]=UAS-sgg.A81T}MB30/rh1>gal4 | Thr^849^ (light) | 248 | 143 | 110 | 167.10 | 0.2482 |
|  |  | Thr^849^ (dark) | 6 | 3 | 1 | 3.24 | 0.1018 |
|  |  | Thr^864^ (light) | 100 | - | - | - | - |
|  |  | Thr^864^ (dark) | 20 | - | - | - | - |
| Shaggy | w[1118]; P{w[+mC]=UAS-sgg.Y214F}2/rh1>gal4 | Thr^849^ (light) | 283 | 149 | 137 | 189.40 | 0.1974 |
|  |  | Thr^849^ (dark) | 8 | 3 | 1 | 4.22 | 0.1709 |
|  |  | Thr^864^ (light) | 88 | - | - | - | - |
|  |  | Thr^864^ (dark) | 16 | - | - | - | - |
| Shaggy | w[1118]; P{w[+mC]=UAS-sgg.KK83-84MI}2.1/rh1>gal4 | Thr^849^ (light) | 206 | 164 | 84 | 151.41 | 0.2894 |
|  |  | Thr^849^ (dark) | 15 | 3 | 1 | 6.12 | 0.4668 |
|  |  | Thr^864^ (light) | 88 | - | - | - | - |
|  |  | Thr^864^ (dark) | 27 | - | - | - | - |
| Salt-inducible kinase 3 | y[1] w[67c23]; P{w[+mC] y[+mDint2]=EPgy2}Sik3[EY14354] | Thr^849^ (light) | 127 | - | - | - | - |
|  |  | Thr^849^ (dark) | 4 | - | - | - | - |
|  |  | Thr^864^ (light) | 113 | - | - | - | - |
|  |  | Thr^864^ (dark) | 19 | - | - | - | - |
| Smell impaired 35A | +; P{ry[+t7.2]=lArB}smi35A[1]; ry[506] | Thr^849^ (light) | 87 | - | - | - | - |
|  |  | Thr^849^ (dark) | 1 | - | - | - | - |
|  |  | Thr^864^ (light) | 121 | - | - | - | - |
|  |  | Thr^864^ (dark) | 8 | - | - | - | - |

| **Kinase/**  **Phosphatase** | **Genotype** | **Phosphory-lation site** | **Phosphorylation in comparison to wild type flies [%]** | | | | |
| --- | --- | --- | --- | --- | --- | --- | --- |
|  |  |  | **1^st^ round** | **2^nd^ round** | **3^rd^ round** | **Mean** | **P-value** |
| SNF1A/AMP-activated protein kinase | SNF1A[1]/FM7i; +; + | Thr^849^ (light) | 247 | 245 | 260 | 250.69 | 0.0010 |
|  |  | Thr^849^ (dark) | 0 | 2 | 1 | 1.14 | <0.0001 |
|  |  | Thr^864^ (light) | 128 |  |  |  |  |
|  |  | Thr^864^ (dark) | 1 |  |  |  |  |
| synaptojanin | w[1118]; PBac{w[+mC]=RB}synj[e02597]/CyO | Thr^849^ (light) | 63 |  |  |  |  |
|  |  | Thr^849^ (dark) | 1 |  |  |  |  |
|  |  | Thr^864^ (light) | 103 |  |  |  |  |
|  |  | Thr^864^ (dark) | 11 |  |  |  |  |
| TGF-β activated kinase 1 | w[*] Tak1[179]; +; + | Thr^849^ (light) | 87 | - | - | - | - |
|  |  | Thr^849^ (dark) | 1 | - | - | - | - |
|  |  | Thr^864^ (light) | 92 | - | - | - | - |
|  |  | Thr^864^ (dark) | 5 | - | - | - | - |
| Tao | w[1118] P{w[+mC]=EP}Tao-1[EP1455]; +; + | Thr^849^ (light) | 101 | - | - | - | - |
|  |  | Thr^849^ (dark) | 1 | - | - | - | - |
|  |  | Thr^864^ (light) | 23 | 35 | 25 | 28.22 | 0.0029 |
|  |  | Thr^864^ (dark) | 2 | 1 | 2 | 1.65 | 0.1042 |
| Target of rapamycin | w[1118]; Mi{ET1}Tor[MB07988]; + | Thr^849^ (light) | 61 | - | - | - | - |
|  |  | Thr^849^ (dark) | 4 | - | - | - | - |
|  |  | Thr^864^ (light) | 111 | - | - | - | - |
|  |  | Thr^864^ (dark) | 1 | - | - | - | - |
